# Changes in LDL Oxidative Status and Oxidative and Inflammatory Gene Expression after Red Wine Intake in Healthy People: A Randomized Trial

**DOI:** 10.1155/2015/317348

**Published:** 2015-05-25

**Authors:** Laura Di Renzo, Luigi Tonino Marsella, Alberto Carraro, Roberto Valente, Paola Gualtieri, Santo Gratteri, Diego Tomasi, Federica Gaiotti, Antonino De Lorenzo

**Affiliations:** ^1^Section of Clinical Nutrition and Nutrigenomics, Department of Biomedicine and Prevention, University of Rome “Tor Vergata”, 00133 Rome, Italy; ^2^Division of Legal Medicine and Social Security, Department of Biomedicine and Prevention, University of Rome “Tor Vergata”, 00133 Rome, Italy; ^3^Department of Surgery and Medical Science, University “Magna Græcia”, 88100 Germaneto, Italy; ^4^Council for Agricultural Research and Experimentation-Viticulture Centre (CRA-VIT), 31015 Conegliano, Italy; ^5^National Institute for Mediterranean Diet and Nutrigenomics (I.N.DI.M.), 87032 Amantea, Italy

## Abstract

Postprandial oxidative stress is characterized by an increased susceptibility of the organism towards oxidative damage after consumption of a meal rich in lipids and/or carbohydrates. Micronutrients modulate immune system and exert a protective action by reducing low density lipoproteins (LDL) oxidation via induction of antioxidant enzymes. We evaluated the gene expression of oxidative stress (HOSp), inflammasome (HIp), and human drug metabolism pathways (HDM) and ox-LDL level at baseline and after the intake of red wine naturally enriched with resveratrol (NPVRW), in association with or without a McDonald's meal (McDM). The ox-LDL levels significantly increase comparing baseline (B) *versus* McDM and decreased comparing McDM *versus* McDM + NPVRW (*P* ≤ 0.05). Percentages of significant genes expressed after each nutritional intervention were the following: (1) B *versus* McDM, 2.88% HOSp, 2.40% of HIp, and 3.37% of HDMp; (2) B *versus* McDM + NPVRW, 1.44% of HOSp, 4.81% of HIp, and 0.96% of HDMp; (3) McDM *versus* McDM + NPVRW, 2.40% of HOSp, 2.40% of HIp, and 5.77% of HDMp; (4) B *versus* NPVRW, 4.80% HOSp, 3.85% HIp, and 3.85% HDMp. NPVRW intake reduced postprandial ox-LDL and the expression of inflammation and oxidative stress related genes. Chronic studies on larger population are necessary before definitive conclusions.

## 1. Introduction

Epidemiological evidence has supported a protective role for diets low in saturated fat and rich in fruits and vegetables as well as a moderate wine consumption against the development and progression of cardiovascular (CVD) and chronic degenerative disease.

The atherosclerotic processes underlying cardiovascular disease are intimately connected with a state of chronic inflammation, involving a variety of pathological changes such as endothelial cell activation, low density lipoprotein (LDL) modification, macrophage chemotaxis, and vessel smooth muscle cell migration [1].

In industrialized societies, LDL cholesterol concentrations often exceed physiological requirements.

This excess in LDL particles promotes transport into the vessel wall where they undergo physicochemical modification, which facilitates their ingress into macrophages resulting in foam-cell formation. It is widely recognized that, together with oxidative stress, vascular inflammation, lipid deposition, and smooth muscle cell differentiation, oxidized low density lipoprotein (ox-LDL) level may play a major role in atherosclerosis and cardiovascular disease, and it is a commonly used marker for oxidative damage [2]. In fact, oxidation of the lipids and apoproteins present in LDL leads to a change in the lipoprotein conformation by which LDL is better able to enter the monocyte-macrophage system of the arterial wall and promote the atherosclerotic process [3]. Moreover, small LDL particles themselves are easily oxidized and detectable in healthy subjects [4]. The oxidative modification of LDL convert the native particles ox-LDL into pathogenic [5], immunogenic, and atherogenic particles [6].

Removal of ox-LDL from circulating blood is a promising therapeutic strategy against atherosclerosis and many other diseases [7].

It is widely accepted that the consumption of fruits and vegetables prevents diseases related to the oxidative processes [8]. For instance, individuals at high cardiovascular risk who improved their diet toward a Mediterranean diet pattern, reached in antioxidants, showed significant reductions in cellular lipid levels and LDL oxidation [9].

Moreover, beneficial effects may partly help to explain the protective CVD effects achieved by foods and beverages containing polyphenols (tea, vegetables, fruits, wine, etc.), that appears to be mediated via a plethora of biochemical pathways and signaling mechanisms, acting either independently or synergistically (e.g., decreasing LDL oxidation, improving endothelial function, increasing nitric oxide release, modulating inflammation and lipid metabolism, and improving antioxidant status) [10]. In particular, the reduction of oxidative stress and inflammation following red wine intake could possibly be due to its polyphenol, and resveratrol, content, as suggested by many studies. Red wine (RW) consumption has shown to prevent the acute impairment of endothelial function that occurs following cigarette smoking or consumption of high fat meal [11] and to modulate monocyte migration in healthy subjects [12]. These effects must be considered independent of some of the protective effects of wine linked to ethanol,* per se*.

It has been suggested that the phenolic content of RW may modulate leukocyte adhesion molecules, whereas both ethanol and polyphenols of RW may modulate soluble inflammatory mediators in high-risk patients [13].

The phytoalexin* trans*-3,5,4′-trihydroxystilbene (*trans*-resveratrol) is present in red grape skins [14] and in wines [15]. Synthesis of resveratrol and its derivatives (*cis*-resveratrol and resveratrol glucoside isomers), called all together stilbenes, is generally formed as natural defense ability of the vines against disease attack.

The content of stilbenes in the final product depends mainly on the grape variety [16], but other factors like canopy management can stimulate the plant's self-defense [17]. Focusing on those practices that can modulate both the qualitative parameters in the classical sense (sugars, acidity, etc.), as well as the most truly innovative and related compounds useful to human health, growing methods can get high quality grapes and at the same time stimulate the stilbene synthesis. Among these the unpruned vineyards can promote the natural presence of stilbenes too [18], whose positive role on human health is known [19].

Although different studies have reported positive data on gene expression after feeding animals with phenolic rich extracts or normal food [20], few human studies have shown the potential use of gene expression profiling in blood leukocytes to study the effects of meal consumption and the intake of red wine from nonpruned vineyard (NPVRW) [21], on gene expression modulation of oxidative stress, inflammatory, and drug metabolism pathway, in association with ox-LDL.

Nevertheless, the nutrigenomic impact of polyphenol in RW has not been extensively investigated [22]. More understanding of the role of NPVRW intake on oxidation of lipoproteins may allow rationally targeted dietotherapy that can beneficially alter postprandial biomarkers associated with CVD.

To our knowledge, no research has compared the acute effects of NPVRW in association with a high fat meal on postprandial biomarkers associated with CVD, including oxidative stress and anti-inflammatory and antioxidant activity.

Therefore, we set up a randomized, crossover, and controlled dietary intervention trial in healthy human volunteers to evaluate and compare the effects on LDL oxidative status after a McDonald meal (McDM), alone or combined with the intake of 30 g alcohol from 250 mL NPVRW.

Moreover, we investigated the gene expression of 208 gene related to oxidative stress, inflammation, and drug metabolism in the same conditions. We hypothesized that the magnitude of changes in postprandial responses would be less with the McDM associated with NPVRW, compared with the McDM alone. This comparison aims to determine if resveratrol is implicated in the nutrigenomic effects of RW.

## 2. Materials and Methods

### 2.1. Study Design

The first aim of the present study was to assess the ox-LDL level in healthy subjects after the intake of 30 g alcohol from 250 mL NPVRW, alone or combined with a McDM. The second outcome was to examine changes of gene expression levels of 75 genes related to oxidative stress pathways (HOSp), 72 of human inflammasome pathways (HIp), and 61 genes of Human Drug Metabolism (HDM) in the same conditions. As control, we repeated the same analysis after the intake of 30 g alcohol from 250 mL NPVRW fasting, with respect to baseline.

The clinical study was a randomized, crossover, and controlled dietary intervention trial. After the enrollment (A), the study was conducted with four intervention arms: (B) baseline; (C) McDM; (D) McDM + 250 mL NPVRW (McDM + NPVRW); (E) 250 mL NPVRW.

Each arm was followed by a three-week washout period to avoid additive effects on treatments to follow. The order of administration was determined using computer-generated random numbers.

**Figure 1:**
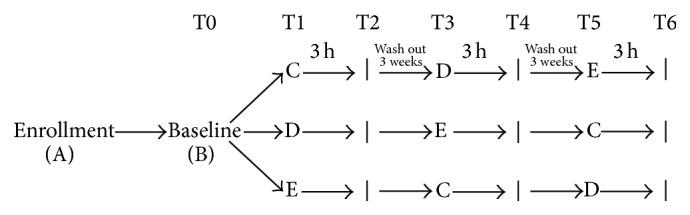
Study design and intervention. The randomized crossover study was divided into 4 nutritional intervention treatments (T0, T1, T3, and T5). The collection of blood sample was detected to 3 hours to each intervention. Between every nutritional intervention, a wash-out period was inserted. In each treatment period subjects consumed (B) baseline (fasting); (C) McDonald's meal; (D) McDonald's meal with 250 mL of not pruned vineyard red wine; (E) 250 mL of not pruned vineyard red wine. Nutrition status was detected at step (A). The blood sample for nutrigenomic and biochemical analysis was collected at the end of each treatment period (T0, T2, T4, and T6).

The experimental study was conducted through the diagram presented in Figure 1.

*Trial Registration*. This protocol has been registered with ClinicalTrials.gov NCT01890070.

### 2.2. Subjects

A total of 30 participants were consecutively recruited from those who participated in routine medical check-up at the Section of Clinical Nutrition and Nutrigenomic, at the University of Rome “Tor Vergata.” All subjects were healthy and had no evidence of chronic disease.

To be eligible for the study, participants had to meet the following inclusion criteria: age between 18 and 65 years, with a BMI ≥ 19 Kg/m^2^. Exclusion criteria were as follows: pregnancy, active smoking, arterial hypertension (≥140/90 mm Hg), body mass index (BMI) > 30 kg/m^2^, acute or chronic diseases, autoimmune disease HIV/AIDS, neoplastic disease, intestinal disorders, vegetarianism, and use of antioxidants or vitamin supplements or any medication that could influence inflammation and oxidative stress. Subjects daily consuming flavonoid-rich beverages, such as tea, herb tea, coffee, cocoa, and fruit juice, more than 500 mL (as estimated from food frequency questionnaire), were also excluded.

Subjects were asked to exclude alcoholic beverages 15 days before the first intervention (run-in period) and during the study. Natural foods rich in antioxidants intake were monitored so that individual diets had similar antioxidant contents throughout the study.

At baseline, all participants were evaluated in terms of their health status. The clinical evaluation is based on anthropometric and body composition evaluation, quantification of ox-LDL, and a genomic evaluation of 208 genes belonging to the pathway of oxidative stress, inflammation, and drug metabolism.

Subjects were asked to maintain their usual lifestyle habits and to report any illness or abnormality presented during the study period.

At the end of each arm of the study design, a clinician assessed any adverse effects from the interventions by going through a checklist of symptoms, including bloating, fullness, or indigestion, altered bowel habit, dizziness, and other symptoms that were possibly associated with the interventions. All patients completed the study.

An informed consent was signed by all participants, in accordance with principles of the Declaration of Helsinki.

Nutritional status assessment, genomic analysis, settings and data collection were performed at the Section of Clinical Nutrition and Nutrigenomic, Department of Biomedicine and Prevention of the University of Rome “Tor Vergata.”

#### 2.2.1. Anthropometric Measurements

After a 12-hour overnight fast, all subjects underwent anthropometric evaluation. Anthropometric measurements for all participants according to standard method were carried out [23]. All the individuals were instructed to take off their clothes and shoes before undergoing the measurements.

Waist and hip measures were taken using a flexible steel metric tape to the nearest 0.5 cm, with subjects standing with arms relaxed by their side and balanced on both feet. The tape was held tight to the skin but without compression of tissue. Hip circumference was also measured according to International Society for the Advancement of Kin anthropometry protocol taken at the greatest posterior protuberance of the buttocks. Waist circumference was measured just above the iliac crest as recommended in the National Institute of Health Guidelines. Body weight (kg) [24] was measured to the nearest 0.1 kg, using a balance scale (Invernizzi, Rome, Italy). Height (m) was measured using a stadiometer to the nearest 0.1 cm (Invernizzi, Rome, Italy). Body mass index (BMI) was calculated using the formula: BMI = body weight/height^2^ (kg/m^2^).

#### 2.2.2. Dual X-Ray Absorptiometry (DXA)

Body composition analysis was assessed by DXA (i-DXA, GE Medical Systems, Milwaukee, WI, USA), according to the previously described procedure, for evaluating soft tissues, that is, total body fat (TBFat) and total body lean (TBLean) [25].

The technique combined a total body scanner, an X-ray source, an internal wheel to calibrate the bone mineral compartment, and an external lucite/aluminium phantom to calibrate the fat compartment. Standard DXA quality control and calibration measures were performed prior to each testing session. The subjects were instructed not to exercise within 24 h from the test. The subjects were given complete instructions on the testing procedure. Individuals were asked to remove all clothing except for undergarments including shoes, socks, and metal items prior to being positioned on the DXA table. Scans were performed with individuals in a supine position. The entire body was scanned beginning from the top of the head and moving in a rectilinear pattern down the body to the feet. The average measurement time was 20 min. The effective radiation dose from this procedure is about 0.01 mSv. The coefficient of variation (coefficient of variation = 100 × SD/mean) intra- and intersubjects ranged from 1% to 5%. The coefficient of variation for bone measurements is less than 1%; coefficient of variation on this instrument for five subjects scanned six times over a nine-month period was 2.2% for TBFat and 1.1% for TBLean.

Total body fat percentage (PBF) was calculated as TBFat mass divided by total mass of all tissues, considering also the total body bone (TBBone), as follows:

(1) $$\begin{array}{c} \text{PBF}=\left(\text{TBFat}+\text{TBLean}+\text{TBBone}\right)\times 100. \end{array}$$

### 2.3. Analysis of Blood Samples

Blood samples (10 mL) were collected into sterile tubes containing EDTA (Vacutainer). All materials were immediately placed on ice and plasma was separated by centrifugation at 1600 ×g for 10 min at 4°C. Laboratory tests included fasting glucose, HDL-cholesterol, LDL-cholesterol, triglycerides, aspartate aminotransferase (AST), alanine transaminase (ALT), creatinine, fibrinogen, and C-reactive protein (CRP) levels and were recorded at baseline. All clinical chemistry analyses, except plasma glucose, serum lipid, CRP, and triglycerides analysis, were carried out with an ADVIA 1800 Chemistry System (Siemens Healthcare), according to standard procedures [26]. Plasma glucose concentrations were measured using the glucose oxidase method with an automated glucose analyzer (COBAS INTEGRA 400, Roche Diagnostics, Indianapolis, IN, USA); serum lipid profile components were determined by standard enzymatic colorimetric techniques (Roche143 Modular P800, Roche Diagnostics, Indianapolis, IN). Serum CRP was measured by a high-sensitivity sandwich enzyme immunoassay from Immundiagnostik. Serum triglycerides were measured on the Beckman Synchron LX20 automated system by a coupled enzymatic method that produces a red-colored complex. All tests were performed using the same lot of reagents or assay plates in order to minimize variability due to differences in reagent lots.

Analyses were carried out at the accredited Clinical Chemical Laboratories of the “Tor Vergata” Hospital of Rome, Italy.

### 2.4. Sample Collection and RNA Extraction

Blood sample was collected and stabilized in PAXgene Blood RNA Tubes (Pre AnalytiX Qiagen, Hombrechtikon, Switzerland) and stored at −80°C until RNA extraction. The total RNA of each collected sample was purified using the PAXgene Blood miRNA Kit according to the manufacturer's instructions (PreAnalytix Qiagen, Hombrechtikon, Switzerland). Aliquots of total RNA were then quantified and assessed for quality by spectrophotometry (Nanodrop, Wilmington, USA) and agarose gel electrophoresis.

### 2.5. Quantitative Real Time PCR and Data Analysis

We used specific RT2 Profiler PCR Arrays (Qiagen, Netherlands): we focused on the Human Oxidative Stress (PAHS-065ZA, Qiagen, Netherlands) pathway, Human Inflammation (PAHS-097ZA, Qiagen, Netherlands) pathway, and Human Drug Metabolism (PAHS-002A, Qiagen, Netherlands) pathway.

We used a specific RT2 Profiler PCR Arrays (Qiagen, Netherlands). Each qRT-PCR experiment was performed in triplicate and repeated at least twice, according to manufacturer's instruction (Qiagen, Netherlands).

The value used to plot relative gene expression was determined using the expression fold change (FC) = 2^−ΔΔCT^ [27]. Raw data were filtered for genes that were significantly changed above factor 1.0 within the 95% confidence interval (*P* ≤ 0.05) for each experiment. Finally, only genes with an absolute FC value of at least ±1.5 and *P* value ≤ 0.05 (indicating a statistical significance) were considered as differentially expressed genes.

### 2.6. Low Density Lipoprotein Oxidative Status

A fasting blood samples were collected and stabilized in EDTA and stored at −80°C until analysis.

Circulating ox-LDL level in plasma was measured by enzyme linked immunosorbent assay using the mAb-4E6 antibody (Mercodia AB, Uppsala, Sweden), according to the customer protocol.

### 2.7. Production, Composition, and Stilbenes Content in Wines

The agricultural trial was started in 2012 on 12-year-old commercial vineyards, located in Ponte di Piave, Treviso, (north-east Italy, 45°43′02.93′′N, 12°32′8:35′′ E, altitude 3 m asl). The variety was Cabernet Sauvignon, grafted on 1103 P, and the vineyard has a density of 3300 plants per hectare. The adopted training system was the free cordon (a permanent spur cordon pruning with no wire above the cordon); “no pruning” (NP) treatment have been provided. For the NP treatment the vines have not undergone cutting, consecutively, for 7 years. A randomized block design was applied, with 3 plots, each one consisting of 2 rows (one per treatment). Surveys were carried out on 15 contiguous vines within the selected rows.

Fifteen vines per treatment were chosen and replicated 3 times; the production of grapes was weighed, and the average weight of the bunch was made; on a sample of berries the polyphenol content, total and extractable, according to the methodology proposed by Di Stefano and Cravero [28], soluble solids content (°Brix), total acidity (TA), and pH were determined. NP was obtained by microvinification of 300 kg of grapes. The contents of some stilbenes, resveratrol,* trans* and* cis*, and* trans*-piceid were determined according to the methodology proposed by Sun et al. [29].

### 2.8. Dietary Treatment Description and Bromatological Composition of Meal

The selected dietary treatment was a high fat meal represented by a McDonald's meal (McDM) consisting in number 1 Big Tasty Bacon and number 1 small French Fries (26.8% of total Kcal from carbohydrates; 18.2% of total Kcal from protein, of which about 70% was comprised of animal proteins; 55% of total Kcal from total fat).

The bromatological composition of McDM was obtained using diet analyser software package Dietosystem (DS Medica SRL, Milan, Italy, version 12.00.13.).

The quality indices of the meal were calculated as follows.

Atherogenic Index (AI), a parameter to determine the atherogenic risk of a diet, was calculated by the ratio between saturated (C14 : 0, C16 : 0, C18 : 0) and unsaturated fat acids (monounsaturated fatty acid (MUFA) and polyunsaturated fatty acid (PUFA)):

(2) $$\begin{array}{c} \text{IA}=\frac{\left[\left(4\times \text{C}14\text{ : }0\right)+\text{C}16\text{ : }0+\text{C}18\text{ : }0\right]}{\left[\Sigma \text{MUFA}+\Sigma \text{PUFA}-\omega 6+\Sigma \text{PUFA}-\omega 3\right]}. \end{array}$$

Thrombogenicity Index (TI) was calculated by the ratio between saturated fat acids (C14 : 0, C16 : 0, C18 : 0) and the sum of MUFA and PUFA:

(3) IT=C14 : 0+C16 : 0+C18 : 0·0.5×MUFA+0.5×PUFA−ω6-fatty  acidsω3-fatty  acidsPUFA +3×PUFA−ω3  fatty  acids+PUFA −ω3-fatty  acidsPUFA−ω6-fatty  acids−1.

Cholesterol/saturated fatty acids Index (CSI) can be used as a fast and accurate way to assess the content of saturated fat and dietary cholesterol. The value of the CSI is expressed on a scale from 1 to 100. The potential atherogenic risk of food referred to the cholesterol and saturated fat contained in the meal; lower index suggests a low probability of incidence of cardiovascular disease:

(4) CSI=1.01×g  of  saturated  fat+0.05×mg  of  cholesterol.

### 2.9. Statistical Analysis

A paired *t*-test or a nonparametric Wilcoxon test was performed to evaluate differences before and after nutritional intervention. In all statistical tests performed, the null hypothesis (no effect) was rejected at the 0.05 level of probability.

The differences between ox-LDL levels in the intervention arms were calculated as follows.

Δ% = ((*Z* − *Y*)/*Y*) × 100, where Δ% is the percentage variation of oxidation of LDL, calculated as ratio of absolute variation to the base value; in particular *Z* is the value of oxidation LDL after nutritional intervention (NPVRW, McDM, and McDM + NPVRW) and *Y* is the value of oxidation if LDL at the nutritional intervention of reference (B and McDM).

The value used to plot relative gene expression was determined using the expression fold change (FC) = 2^−ΔΔCT^ [27].

Data were normalized and we have considered an absolute fold change at least equal to ±2.00 (absolute FC ≥ ±2.00).

Transcripts were declared differently expressed only when *P* < 0.05 and fold changes were either <0.8 (downregulated) or >1.2 (upregulated), as previously described [21].

## 3. Results

### 3.1. Quality Parameters of Wine

The NPVRW treatment reported the following quality parameters: the soluble solids (°Brix) equal to 19.20, a titratable acidity equal to 7.4 g/L, and a pH as 3.38. Regarding the content flavonoids and anthocyanins, the wine showed a content of total anthocyanins equivalent to 541 mg/Kg of berry, a content of extractable anthocyanins equal to 317 mg/Kg of berry. Total flavonoids were detected equal to 1346 mg/Kg and a content of extractable flavonoids equivalent to 575 mg/Kg of berry.

### 3.2. Bromatological Composition of Dietary Treatment and Indices

The macronutrient content of McDM was 1144.04 kcal, 81.89 g of carbohydrates, 51.99 g of proteins (40.78 g of animal proteins, 10.60 g of vegetal proteins), 68.89 g of fats (of which 21.71 g saturates, 30.71 g monounsaturates, and 13.96 g unsaturates), and 3.68 g of dietary fiber (of which 1.51 g soluble, and 2.15 g insoluble fiber). The quality indices of McDM were 1.97 of AI, 1.73 of TI, and 31.05 of CSI.

### 3.3. Clinical Trial

Of the 30 initial participants initially enrolled, 24 subjects were eligible for the study. Four subjects declined to participate during the first phase, and another two did not meet the inclusion criteria: one of them measured a BMI > 25 Kg/m^2^, one had a history of heart attack. In Tables 1 and 2 the nutritional status characteristics and blood parameters of subjects are shown, before nutritional intervention. The value of obese subjects was obtained by DXA: total percentage of obese was estimated as a 58.33% of total subjects: 71.43% of female and 40.00% of men. According to BMI, no obese subjects were identified. None of the subjects were osteoporotic. According to ASMMI, 16.67% of total subjects were sarcopenic: 20.00% of male and 14.29% women were sarcopenic.

**Figure 2:**
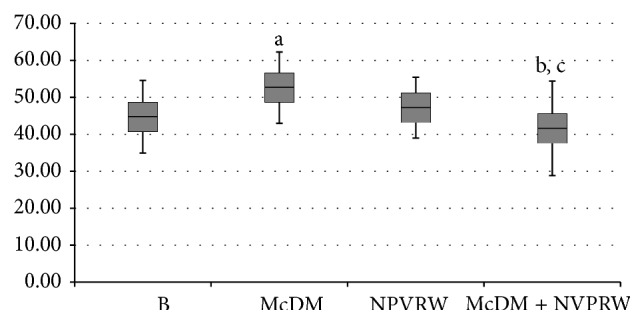
Comparative values of ox-LDL level for each treatment intervention. The significant values are expressed as (a) baseline* versus* McDonald's meal (*P* ≤ 0.05); (b) McDonald's meal* versus* McDonald's meal + not pruned vineyard red wine (*P* ≤ 0.05); (c) baseline* versus* not pruned vineyard red wine (*P* > 0.05); baseline* versus* McDonald's meal + not pruned vineyard red wine (*P* > 0.05).

The comparison of ox-LDL level in the nutritional intervention was shown in Figure 2. A significant increase (*P* ≤ 0.05) of ox-LDL level was observed between B andMcDM, showing a Δ% = 17.56%. The ox-LDL levels significantly decreased (*P* ≤ 0.05) comparing McDM* versus* McDM + NPVRW (Δ% = −20.94%). No significant value (*P* > 0.05) was highlighted in B* versus* McDM + NPVRW (Δ% = −7.06%) and in B* versus* NPVRW (Δ% = 5.48%).

**Figure 3:**
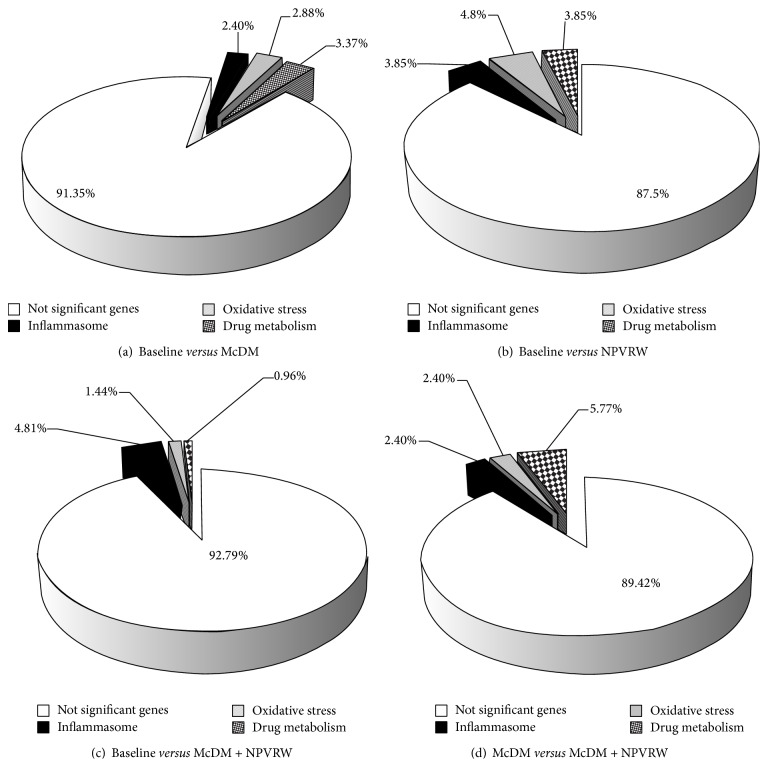
Expression of genomic pathways. Percentage of significant genes in comparison between: (a) baseline* versus* McDonald's meal; (b) baseline* versus* NPVRW; (c) baseline* versus* McDonald's meal + not pruned vineyard red wine; (d) McDonald's meal* versus* McDonald's meal + not pruned vineyard red wine.

Moreover, we analyzed a total of 208 genes after each nutritional intervention. In particular, we focused on different expression levels of 75 genes of oxidative stress, 72 genes of inflammasome and 61 genes of Human Drug Metabolism pathways (see supplement Table S1–3 in Supplementary Material available online at http://dx.doi.org/10.1155/2015/317348). The different fold change levels were analyzed for the condition: (i) B* versus* McDM; (ii) B* versus* NPVRW; (iii) B* versus* McDM + NPVRW; (iv) McDM* versus* McDM + NPVRW. After data normalization, considering an absolute fold change at least equal to ±2.00 (absolute FC ≥ ±2.00), we identified differential gene expression levels for each condition, as shown in Figure 3.

**Figure 4:**
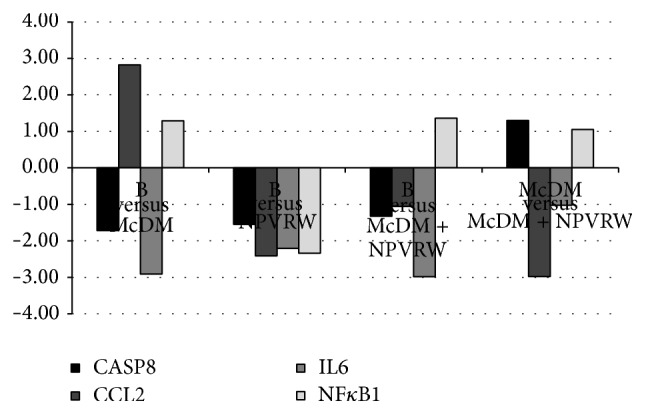
Gene expression in each dietary intervention for inflammasome pathway. Different levels of up- and downregulation of genes analyzed between McDonald's meal andMcDonald's meal with not pruned vineyard red wine. The significant values are expressed as *P* ≤ 0.05 for a level of fold change >±2.00. Inflammasome genes:* CASP8*, Caspase 1 apoptosis related cysteine peptidase (NM_1228);* CCL2*, chemokine (C-C motif) Ligand 2 (NM_2982);* IL6*, Interleukin 6 (NM_600);* NFKB1*, Nuclear Factor of Kappa Light Polypeptides Gene Enhancer in B-Cells 1 (NM_3998).

**Figure 5:**
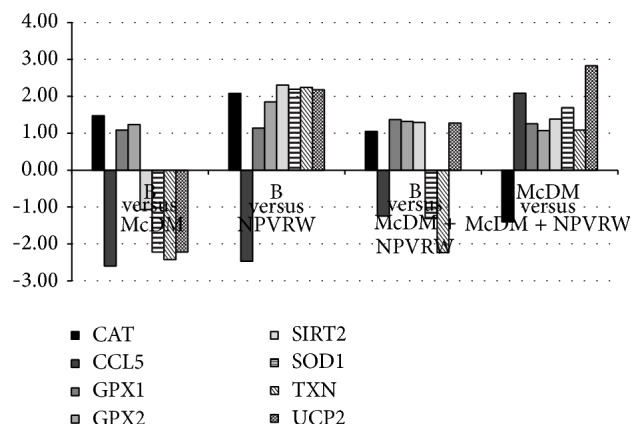
Gene expression in each dietary intervention for oxidative stress pathway. Different levels of up- and downregulation of genes analyzed between McDonald's meal andMcDonald's meal with not pruned vineyard red wine. The significant values are expressed as *P* ≤ 0.05 for a level of fold change > ±2.00. Oxidative stress genes:* CAT*, Catalase (NM_1752);* CCL5*, Copper Chaperone for Superoxide Dismutase (NM_2985);* GPX1*, Glutathione Peroxidase 1 (NM_581);* GPX2*, Glutathione Peroxidase 1 (gastrointestinal) (NM_2083);* SIRT2*, Sirtuin 2 (NM_12237);* SOD1*, Superoxide Dismutase 1 Soluble (NM_454);* TXN*, Thioredoxin (NM_3329);* UCP2*, Uncoupling Protein 2 (Mitochondrial Proton Carrier) (NM_3355).

**Figure 6:**
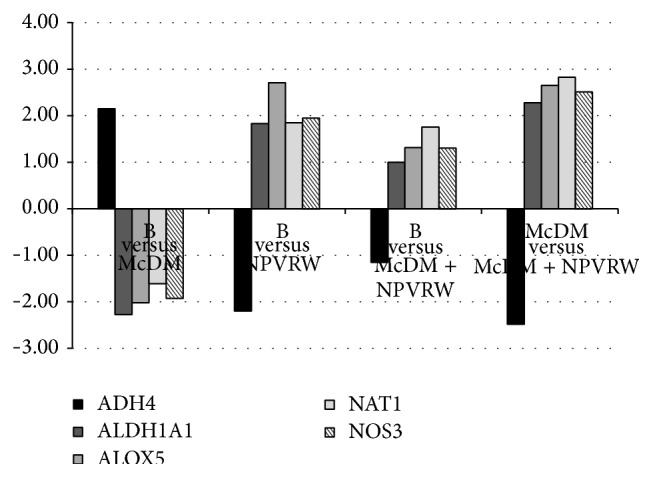
Gene expression in each dietary intervention for Human Drug Metabolism pathway. Different levels of up- and downregulation of genes analyzed between McDonald's meal andMcDonald's meal with not pruned vineyard red wine. The significant values are expressed as *P* ≤ 0.05 for a level of fold change ±2.00. Human Drug Metabolism genes:* ADH4*, Alcohol Dehydrogenase 4-Class 2 Pi Polypeptide (NM_670);* ALDH1A1*, Aldehyde Dehydrogenase 1 Family Member A1 (NM_689);* ALOX5*, Arachidonate 5-Lipoxygenase (NM_698);* NAT1*, N-Acetyltransferase 1 (Arylamine N-Acetyltransferase) (NM_662);* NOS3*, Nitric Oxide Synthase 3 (Endothelial Cell) (NM_603).

In Figures 4, 5, and 6 we showed the most representative genes of the studies pathways; a fold change >±2.00 was considered significant (*P* ≤ 0.05).

In Figure 4 genes of inflammasome pathway are reported, comparing: (i) B* versus* McDM; (ii) B* versus* NPVRW; (iii) B* versus* McDM + NPVRW; (iv) McDM* versus* McDM + NPVRW: CASP8, Caspase 1 Apoptosis Related Cysteine Peptidase (NM_1228); CCL2, Chemokine (C-C motif) Ligand 2 (NM_2982); IL6, – Interleukin 6 (Interferon Beta 2) (NM_600); NFKB1, Nuclear Factor of Kappa Light Polypeptides Gene Enhancer In B-Cells 1 (NM_3998).

In Figure 5 genes of oxidative stress pathway are reported, comparing (i) B* versus* McDM; (ii) B* versus* NPVRW; (iii) B* versus* McDM + NPVRW; (iv) McDM* versus* McDM + NPVRW:* CAT*, Catalase (NM_1752);* CCL5*, Copper Chaperone for Superoxide Dismutase (NM_2985);* GPX1*, Glutathione Peroxidase 1 (NM_581);* GPX2*, Glutathione Peroxidase 1 (gastrointestinal) (NM_2083);* SIRT2*, Sirtuin 2 (NM_12237);* SOD1*, Superoxide Dismutase 1 Soluble (NM_454);* TXN*, Thioredoxin (NM_3329);* UCP2*, Uncoupling Protein 2 (Mitochondrial Proton Carrier) (NM_3355).

In Figure 6 genes of Human Drug Metabolism pathway are reported, comparing (i) B* versus* McDM; (ii) B* versus* NPVRW; (iii) B* versus* McDM + NPVRW; (iv) McDM* versus* McDM + NPVRW:* ADH4*, Alcohol Dehydrogenase 4-Class 2 Pi Polypeptide (NM_670);* ALDH1A1*, Aldehyde Dehydrogenase 1 Family Member A1 (NM_689);* ALOX5*, Arachidonate 5-Lipoxygenase (NM_698);* NAT1*, N-Acetyltransferase 1 (Arylamine N-Acetyltransferase) (NM_662);* NOS3*, Nitric Oxide Synthase 3 (Endothelial Cell) (NM_603).

In the supplementary tables the fold change of all analyzed genes and related statistical significance were reported (see Tables S1–3).

## 4. Discussion

Dietary patterns have been associated with several cardiovascular risk factors such as blood pressure, obesity, serum lipids, and inflammatory markers [30].

In postprandial state, cellular structures such as proteins, carbohydrates, nucleic acids, and lipids are damaged by oxidative processes [31]. High fat diets are contributory atherosclerosis risk factors, since it can cause an acute modification in endothelial function. This effect begins two hours after food intake and continues for several hours.

Considering that after each meal, blood concentrations of glucose and lipids are raised, and this postprandial increase lasts for a rather long time; these changes might be of importance in the process of atherosclerosis initiation and progression [32]. Moreover, after each meal the production of reactive oxygen species (ROS) will lead to the activation of factor nuclear factor-*κ*B (NF-KB). This factor is a transcription factor which is contributed in immunity, inflammation, and regulation of cell proliferation, growth, and apoptosis by controlling the expression of many genes [33]. These are all among the mechanisms that might be contributed in the progression of atherosclerosis [34].

Although native LDL is exposed to all enzymatic and nonenzymatic oxidants, they are protected by a potent array of antioxidants in plasma. Moreover, some of these antioxidants are a part of the LDL composition. The LDL and other biomolecules are protected from free radical attack by the action of antioxidant capacity in the blood. The protective effects of enzymatic and nonenzymatic antioxidant activity are exerted in a network manner. Therefore, plasma antioxidant status is the result of interaction and cooperation of various antioxidants [35].

A decrease in ox-LDL was reported after consumption of beverage as source of polyphenols, such as grape juice, red wine, green tea, and cocoa drink and cranberries [36, 37].

Micronutrients modulate immune system and exert a protective action by reducing LDL-cholesterol oxidation via induction of antioxidant enzymes [38].

According Setorki et al. using high-dose vinegar with cholesterolemic induced a significant decrease in ox-LDL compared to hypercholesterolemic diet [39].

Natella et al. have demonstrated that grape seed proanthocyanidins with meal can lower the postprandial oxidative stress through decreasing oxidants concentration and increasing the level of serum antioxidants. These effects can eliminate the oxidative modification of LDL [40].

Moreover, according to observational studies [41], due to the synergism of different nutrients consumed in a daily diet, it could be difficult to separate out specific effects. However, it was of our interest investigating how polyphenols from RW intake, combined with McDM, could be associated with circulating markers of oxidative stress and inflammation, then contributing to increase cardiometabolic risk.

In our study, NPVRW had a notably higher amount of* trans*-resveratrol and other stilbenes content compared to commercial wine. This might be an indication that less invasive pruning methods (like the extreme case of no pruning) can lead to a higher amount of resveratrol.

According to our previous data [21], in the present study, after the consumption of McDM with NPVRW compared to McDM, we observed a significant decrease of ox-LDL levels, with protective effect towards LDL oxidation. The opposite is observed for the McDM compared to B. Moreover, no significant effect on ox-LDL was highlighted after NPVRW intake.

The ox-LDL level, combined with transcriptomics analysis gives us further insights to understand the global effect of dietary patterns on health. In this study, McDM was related to biological pathways associated with oxidative stress, inflammatory, and drug metabolism response.

Opposite to NPVRW intake effect, after consumption of McDM and McDM + NPVRW, we observed an upregulation of NF-*κ*B-1 gene expression, probably due to the consumption of a high fat meal. NF-*κ*B is the master regulator of the immune/inflammatory response; by regulating NF-*κ*B, Sirtuin-1 (SIRT-1) may inhibit the expression of genes involved in inflammation that is directly related with the NF-*κ*B signaling pathway [42]. In addition, an increasing number of studies suggest that resveratrol plays an important role in protecting mitochondria and attenuating mitochondrial ROS production, improving endothelium function, inhibiting inflammatory processes and decreasing the rate of endothelial apoptosis [43]. Activation of the redox-sensitive NF-*κ*B is believed to be an important component of the cascade of events triggered by psychosocial stress, leading to inflammation, thrombosis, and vascular damage. NF-*κ*B subunits are expressed ubiquitously and can be activated by a wide range of stimuli, such as ROS, cytokines, infection, and DNA damage; however, their actions are regulated in a cell- and stimulus-specific manner, leading to a diverse spectrum of effects [44].

**Figure 7:**
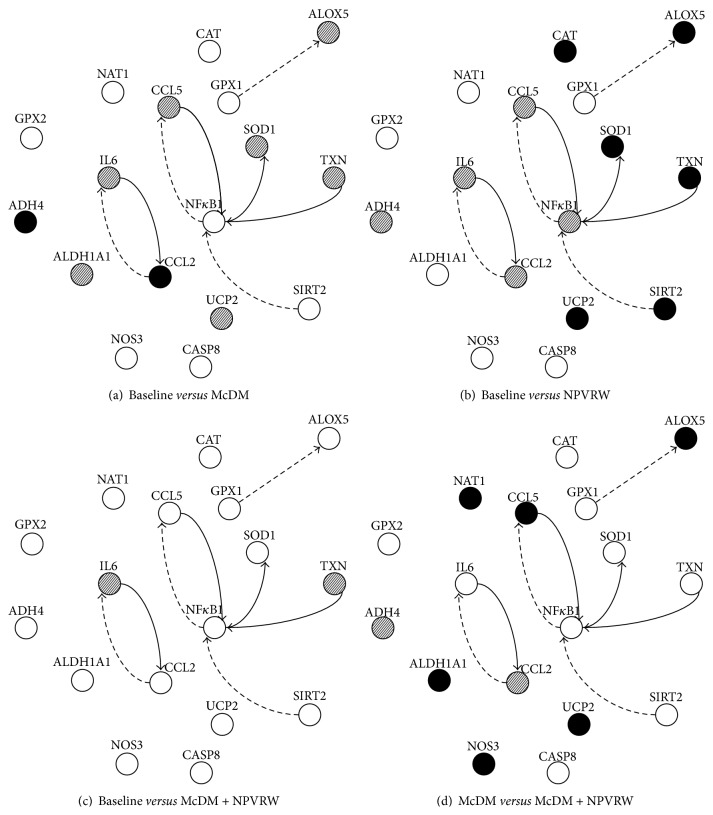
Nutrigenomic networking. Network showing upregulation (solid arrows) and downregulation (dashed arrows) of genes involved in inflammation, oxidative stress, and drug metabolism, in comparison with (a) baseline* versus* McD, (b) baseline* versus* NPVRW, (c) baseline* versus* McD + NPVRW, and (d) McD* versus* McD + NPVRW. Filled circle indicates upregulation in response to treatment; dashed circle indicates downregulation in response to treatment; empty circle indicates not significant genes.* Oxidative stress genes*:* CAT*, Catalase (NM_1752);* GPX1*, Glutathione Peroxidase 1 (NM_581);* GPX2*, Glutathione Peroxidase 1 (gastrointestinal) (NM_2083);* UCP2*, Uncoupling Protein 2 (Mitochondrial Proton Carrier) (NM_3355);* SOD 1*, Superoxide Dismutase 1 Soluble (NM_454);* NOX4*, Nadph. Oxidase 4 (NM_16931);* ALB*, Albumin (NM_477);* CCL5*, Copper Chaperone for Superoxide Dismutase (NM_2985).* Inflammasome genes*:* CASP8*, Caspase 1 Apoptosis Related Cysteine Peptidase (NM_1228);* NFKB1*, Nuclear Factor of Kappa Light Polypeptides Gene Enhancer in B-Cells 1 (NM_3998);* CCL2*, Chemokine (C-C motif) Ligand 2 (NM_2982);* PYCARD*, Pyd and Card Domain Containing (NM_13258);* RPLP0*, Ribosomal Protein Large P0 (NM_1002); NLRP12, Nrl Family Pyrin Domain Containing 12 (NM_33297); IL 6, Interleukin 6 (Interferon Beta 2) (NM_600).* Human Drug Metabolism genes*:* NAT1*, N-Acetyltransferase 1 (Arylamine N-Acetyltransferase) (NM_662);* NOS3*, Nitric Oxide Synthase 3 (Endothelial Cell) (NM_603);* APOE*, Apolipoprotein E (NM_41);* ADH4,* Alcohol Dehydrogenase 4-Class 2 Pi Polypeptide (NM_670);* ALOX15*, Arachidonate 15-Lipoxygenase (NM_1140);* ALOX5*, Arachidonate 5-Lipoxygenase (NM_698);* HSD17 B2*, Hydroxysteroid (17 Beta) Dehydrogenase 2 (NM_2153);* ALDH1A1*, Aldehyde Dehydrogenase 1 Family Member A1 (NM_689);* MT2A*, Metallothionein 2a (NM_5953).

Sirtuin family seems to mediate some of the effects of dietary restriction [45]. Sirt 1, activated through both calorie restriction and resveratrol [46], mediates the beneficial impact of each on longevity and health [47], acting by deacetylating transcription factors, such as the tumor suppressor p53, the Forkhead box (FOX) family of transcription factors FOXO1, FOXO3, and FOXO4 and the transcription factors NF-*κ*B [48]. SIRT2 modulates ROS production and increases resistance to oxidative stress [49]. It has therefore been suggested that resveratrol mimics the protective effects of dietary restriction, since it can activate the mammalian sirtuin* in vitro* assays [50]. We observed different levels of SIRT2 expression after consumption of the NPVRW and McDM + NPVRW, compared to B, probably owed to the resveratrol present in red wine, as suggested by Schirmer et al. [51].  Figure 7 shows the network of these nutrigenomic pathways.

We have observed an upregulation of Alcohol Dehydrogenase (ADH4) in comparison between B and McDM meal. Probably, this is due to the presence of ethanol in bread of McDM sandwich; in fact in the other condition, ADH4 is downregulated and Aldehyde Dehydrogenase (ALDH1A1) is upregulated, according to metabolic pathway of ethanol [52].

Monocyte chemoattractant protein-1 (MCP-1), also known as chemokine (C-C motif) Ligand 2 (CCL2), is a proinflammatory chemokine and recruits and activates monocytes during the inflammatory response. Our results, from the comparison between B and McDM, suggest that CCL2 inhibits the transcription of IL-6, according to Mandrekar et al. [53], who highlighted the role of CCL2 in the regulation of TNF-*α* and IL-6. However, the comparison between B* versus* NPVRW, B* versus* McDM + NPVRW, and McDM* versus* McDM + NPVRW shows a downregulation for each gene, CCL2 and IL-6, highlighting a control over inflammation genomic condition. This is probably due to the presence of phytochemical of NPVRW.

As previously demonstrated [21], we have underlined the relation between Catalase (CAT) and CCL5. In a proinflammatory situation, due to the consumption of high fat meal, CAT downregulated the expression of CCL5. On the other hand, comparing McDM* versus* McDM + NPVRW we obtained a downregulation of CAT and an upregulation of CCL5. The positive effect of wine is highlighted in comparison between B and NPVRW, such as in comparison between McDM and McDM + NPVRW, in which TXN is upregulated. In fact Thioredoxin is critical for redox regulation of NF-*κ*B [20] and it has general intracellular antioxidant activity and when upregulated or overexpressed protects against oxidative stress [54].

According to Poulsen et al. our results show an UCP2 upregulation in B* versus* NPVRW, B* versus* McDM + NPVRW, and McDM* versus* McDM + NPVRW comparison. We concluded that NPVRW, in association with a high fat meal, could improve the upregulation of UCP2 preventing mitochondrial oxidative stress and hepatic fat accumulation [55].

Our results show a downregulation of Thioredoxin (TXN) gene in B compared to McDM and a downregulation after McDM + NPVRW of the same gene. TRX or TXN is a 12 Kda multifunctional protein with a redox-active site (Cys-Gly-Pro-Cys), which is involved in dithiol/disulfide exchange reaction. Furthermore, reduced TRX prevents apoptosis via an inhibitory binding to apoptosis signal-regulating kinase 1 (ASK-1), whereas this binding is lost when TRX is oxidized [56]. An increased TRX expression could help to increase the reduction of intracellular proteins and other biomolecules as part of the antioxidant defense [57].

Our results suggest that downregulation of TXN gene may be due to hazelnut supplementation but still it is still unclear what the interaction processes are.

However, these data reflect gene expression profile and statistical predictions and need to be confirmed by further research.

Previous study reported that a simple change of ≈25% of energy load from fat to carbohydrate in a meal significantly improves postprandial proatherogenic factors [58]. Results observed in this exploratory study support the scientific evidence regarding the deleterious impacts of a high fat meal, such as the McDM, which represents a usual meal of Western dietary pattern. Moreover, our results also indicate that expression of genes in pathways related to chronic disease are positively influenced by the enrichment of polyphenol during the meal, as demonstrated by the effects of NPVRW intake.

A limit of the study was due to the small number of participants, although for genomic studies a numerosity of 8 to 25 subjects is acceptable [21]. In addition, it would be appropriate to evaluate the impact on gene expression of other factors associated with a healthy or unhealthy lifestyle, as the physical activity [59].

Moreover, another limit of the study was the short-term treatment, due to high level of quality indexes of McDM, such as AI, CSI, and TI that are referred to a higher amount of saturated fat and related to oxidized LDL and pathogenic factor [60].

Since the biomarkers used in our study are not commonly examined in the clinical setting, for example, gene expression of drug metabolism, inflammation, and oxidative stress, our results can only lead to limited conclusions. However, as previously reported [21], our data highlighted the positive effect of NPVRW intake alone or in association with McDM on ox-LDL, indicating that the antioxidant potential of the nutrients found in red wine may be an essential component to combatting chronic noncommunicable diseases linked to inflammation and oxidative stress.

Although more studies are recommended to investigate the role of antioxidant capacity on LDL oxidation and gene expression, our study confirms the need of a long term lifestyle intervention based on healthy diet, combined with a moderate intake of high quality red wine, to improve mechanisms of metabolic and cardiovascular diseases, such as oxidative stress and inflammation [61]. In fact, whereas excessive alcohol consumption is among the leading contributors to morbidity and mortality worldwide [62], moderate alcohol consumption (2 drinks/day for men and 1 drink/day for women) may have some health benefits [61].

In conclusion, the idea that is possible to reduce the cardiovascular stress due to postprandial oxidative stress over the course of a meal may seem trivial; however, these daily insults over time may lead to CVD. Consuming RW may be a manner to reduce the magnitude of these daily insults, which potentially will reduce CVD risk.

The results from this study demonstrate that consuming red wine, naturally enriched with resveratrol, results in lower postprandial ox-LDL concentration and lower inflammation, according to the vision of social medicine, for the prevention and control of CVD and chronic degenerative diseases. Although the present data do not allow the conclusion that NPVRW intake has an acute protective value for atherosclerosis, it seems reasonable to conclude that these results show the favorable acute effects on some risk factors of atherosclerosis, such as ox-LDL, and overexpression of inflammation and oxidative stress related genes. However, more research is needed on a larger population and on chronic effects of NPVRW intake before definitive conclusions can be made.

## Supplementary Material

The supplementary tables report different expression levels, expressed as fold changes (FC) of 75 genes of oxidative stress, 72 genes of inflammasome, and 61 genes of Human Drug Metabolism pathways. Genes with an absolute FC value of at least ±1.5 and *P* value ≤ 0.05 (indicating a statistical significance) were considered as differentially expressed genes.

## Figures and Tables

**Table 1:** Baseline characteristics of healthy volunteers.

Parameters	Min, max	Mean ± SD
Age	25,60–46,90	31,04 ± 5,89
Height	158,00–186,00	169,58 ± 10,97
Weight	48,00–92,70	66,16 ± 12,24
BMI	20,00–27,10	23,42 ± 2,51
Neck	33,00–43,00	37,44 ± 3,17
Waist	68,00–86,00	76,44 ± 5,13
Abdomen	78,00–102,00	88,44 ± 8,56
Hips	88,00–108,00	97,50 ± 7,49
W/H	0,67–0,88	0,79 ± 0,06
PBF	18,80–40,90	28,47 ± 6,38
TBF	11,71–28,35	18,72 ± 5,29
TBL	34,18–60,22	44,61 ± 9,48
ASMMI	5,33–8,65	6,90 ± 1,02

Results are expressed in mean value ± standard deviation and minimum and maximum for each parameter.

BMI: body mass index; W/H: waist/hips ratio; PBF: percentage of body fat mass; TBF: total body fat mass; TBL: total body lean; ASMMI: appendicular skeletal muscle mass index.

**Table 2:** Baseline blood values of healthy volunteers.

Blood parameters	Min, max	Mean ± SD
Azotemia	21,00–44,00	32,22 ± 7,29
Creatinine	0,70–1,14	0,92 ± 0,16
Glycemia	80,00–101,00	89,89 ± 6,88
Total cholesterol	128,00–207,00	166,11 ± 22,83
HDL cholesterol	38,00–62,00	48,25 ± 9,36
LDL cholesterol	51,00–132,00	95,13 ± 27,95
Triglycerides	35,00–101,00	63,75 ± 28,04
AST	10,00–37,00	23,13 ± 7,88
ALT	14,00–31,00	22,50 ± 6,05
RCP	0,10–1,00	0,34 ± 0,28
ESR	5,00–13,00	8,38 ± 3,50
Fibrinogen	226,00–300,00	250,83 ± 28,90

Results are expressed in mean value ± standard deviation and minimum and maximum for each parameter. HDL: high density lipoprotein; LDL: low density lipoprotein; AST: aspartate transaminase; ALT: alanine transaminase; PCR: reactive C protein; ESR: erythrocyte sedimentation rate.
