# Supplementary material for: Changes in LDL Oxidative Status and Oxidative and Inflammatory Gene Expression after Red Wine Intake in Healthy People: A Randomized Trial
Source: Mediators Inflamm. 2015 May 25;2015:317348. doi: 10.1155/2015/317348 (PMC4458536; doi:10.1155/2015/317348)
Supplement: Supplementary file 1 — The supplementary tables report different expression levels, expressed as fold changes (FC) of 75 genes of oxidative stress, 72 genes of inflammasome, and 61 genes of Human Drug Metabolism pathways. Genes with an absolute FC value of at least ±1.5 and P value ≤ 0.05 (indicating a statistical significance) were considered as differentially expressed genes. [file 317348.f1.pdf]

Supplement tables

Table1S. Fold Change between Baseline *vs* Mc Donald's meal in Inflammasome genes.

Gene Description	Symbol	Gene Bank (NM)	Fold Change between Baseline and McD (a) *p* <0,05 (b) *p* >0,05
Absent in melanoma 2	AIM2	004833	-1,03 (b)
B-cell ccl/lynphoma 2	BCL2	000633	-1,00 (b)
Bcl2 like 1	BCL2L1	138578	-1,58 (b)
Baculoviral iap repeat containig 2	BIRC2	001166	-1,04 (b)
Baculoviral iap repeat containig 3	BIRC3	001165	1,30 (b)
Caspase recruitment domain family memer 18	CARD18	021571	1,36 (b)
Caspase recruitment domain family memer 6	CARD6	032587	1,16 (b)
Caspase 1 apoptosis related cysteine peptidase (interleukin 1 beta convertase)	CASP1	033292	-1,22 (b)
Caspase 1 apoptosis related cysteine peptidase	CASP5	004347	-1,45 (b)
Caspase 1 apoptosis related cysteine peptidase	CASP8	001228	1,30 (b)
Chemokyna (c-c motive) ligand 2	CCL2	002982	-1,72 (b)
Chemokyna (c-c motive) ligand 7	CCL7	006273	1,36 (b)
Cd 40 ligand	CD40LG	000074	1,66 (b)
Casp8 and fad dlike apoptosis regulator	CFLAR	003879	-1,56 (b)
Conserved helix loop helix ubiquitose kinase	CKUK	001278	-1,50 (b)
Class ii maior histocompatibility complex trans activator	CIITA	000246	-1,16 (b)
Cathepsin b	CTSB	001908	1,13 (b)
Chemokine (cxc motif) ligand 1 (melanome growth stimolating activity alpha)	CXCL1	001511	1,07 (b)
Chemokine (cxc motif) ligand 2	CXCL2	002089	1,07 (b)
Fas (tnfrsf6) asociated via death domain	FAD D	003824	-1,13 (b)
Heat shock protein 90 kda alpha (citosolyc) class a member 1	HSP90AA1	001017963	-1,71(b)
Interferon beta 1 fibrobalst	IFNB1	002176	-1,08 (b)
Interferon gamma	IFNG	000919	-1,03 (b)
Inibitor of kappa light	IKBKB	001556	-1,32 (b)

polypeptide gene Enancher in b-cell kinase beta			
Inibitor of kappa light polypeptide gene Enancher in b-cell kinase gamma	IKBKG	003639	-1,09 (b)
Inerleukine 12a (natuarl killer cell stimolatory factor 1 cytotoxic limphocyta maturation factor 1 p35)	IL12A	000882	1,18 (b)
Inerleukine 12b (natuarl killer cell stimolatory Factor 2 cytotoxic limphocyta maturation factor 2 p40)	IL 12B	002187	1,36 (b)
Interlukine 18 (interferon gamma inducuing factor)	IL18	001562	-1,09 (b)
Interleukine 1 beta	IL1B	000576	1,35 (b)
Interleukine 33	IL33	033439	1,36 (b)
Interluekine 6 (interferone beta 2)	IL 6	000600	-2,91 (a)
Interferone regulaotory factor 2	IRF2	002199	1,26 (b)
Mitogen activated protein kinase kinase kinase 7	MAP3K7	003188	-1,17 (b)
Mitogen activated protein kinase 1	MAPK1	002745	-1,48 (b)
Mitogen activated protein kinase 11	MAPK11	002751	-1,09 (b)
Mitogen activated protein kinase 12	MAPK12	002969	1,62 (b)
Mitogen activated protein kinase 13	MAPK13	002754	-1,43 (b)
Mitogen activated protein kinase 3	MAPK3	002746	-1,24 (b)
Mitogen activated protein kinase8	MAPK8	002750	-1,16 (b)
Mitogen activated protein kinase 9	MAPK9	002752	1,39 (b)
Myeloid differentation primary response gene (88)	MYD88	002468	-1,11 (b)
Nuclear factor of kappa light polypeptides Gene enhancer in b-cells 1	NFKB1	003998	1,29 (b)
Nuclear factor of kappa light polypeptides Gene enhancer in b-cells inhibitor alpha	NFKBIA	020529	-1,35 (b)
Nuclear factor of kappa light polypeptides Gene enhancer in b-cells inhibitor beta	NFKBIB	002503	-1,53(b)
Nrl family card domain	NLRC4	021209	-1,32 (b)

containing 4			
Nrl family card domain containing 5	NLRC5	032206	-1,77 (b)
Nrl family pyrin domain containing 1	NLRP1	033004	-1,32 (b)
Nrl family pyrin domain containing 12	NLRP12	033297	2,10 (a)
Nrl family pyrin domain containing 3	NLRP3	183395	1,10(b)
Nrl family pyrin domain containing 4	NLRP4	134444	1,36 (b)
Nrl family pyrin domain containing 5	NLRP5	153447	1,36 (b)
Nrl family pyrin domain containing 6	NLRP6	138329	-1,52 (b)
Nrl family x 1	NLRX1	024618	-1,43 (b)
Nucleotide binding oligomerization Domain containing 1	NOD1	006092	-1,45 (b)
Nucleotide binding oligomerization Domain containing 2	NOD2	022162	1,05 (b)
Purinergic receptor p2x ligand gated ion channel 7	P2RX7	002562	-1,06 (b)
Pannexin 1	PANX1	015368	-1,65 (b)
Phosphoprotein enriched in astrocites 15	PEA15	003768	1,08 (b)
Prolin serin threonine phosphatase Interacting protein 1	PSTPIP1	003978	1,06 (b)
Prostaglandin endoperoxidase synthase 2 (prostaglandin g/h synthase and cyclooxigenase)	PTGS2	000963	-1,65 (b)
Pyd and card domain containing	PYCARD	013258	2,19 (a)
Pyd (pyrin domain) conteing 1	PYCD1	152901	1,36 (b)
Receptor interacting serin threonine kinase 2	RIPK2	003821	-1,30 (b)
Sgt1 suppressor of g2 allele of skp 1 (*s. Cerevisiae*)	SUGT1	006704	-1,01 (b)
Tgf beta activated kinase 1/map 3k7 binding protein 1	TAB1	006116	-1,60 (b)
Tgf beta activated kinase 1/map 3k7 binding protein 2	TAB2	015093	-1,11 (b)
Tool interleukine 1 receptor (tir) domain containing adaptor protein)	TIRAP	001039661	1,03 (b)
Tumor necrosis factor	TNF	000594	1,74 (b)
Tumor necrosis factor	TNFSF11	003701	1,73 (b)

(ligand) superfamily member 11			
Tumor necrosis factor (ligand) superfamily member 14	TNFSF14	003807	1,18 (b)
Tumor necrosis factor (ligand) superfamily member 4	TNFS4	003326	-1,20 (b)
Tnf receptor associated factor 6	TRAF 6	004620	-1,27 (b)
Tioredoxine intercating protein	TXNPI	006472	-1,87 (b)

Table 2S. Fold Change between Baseline *vs* Mc Donald's meal with not pruned vineyard red wine in Inflammasome genes.

Gene Description	Symbol	Gene Bank (NM)	Fold Change between Baseline and McDRW (a) *p* <0,05 (b) *p* >0,05
Absent in melanoma 2	AIM2	004833	-1,18 (b)
B-cell ccl/lynphoma 2	BCL2	000633	-1,14 (b)
Bcl2 like 1	BCL2L1	138578	-2,47 (a)
Baculoviral iap repeat containig 2	BIRC2	001166	-1,28 (b)
Baculoviral iap repeat containig 3	BIRC3	001165	-2,11 (a)
Caspase recruitment domain family memer 18	CARD18	021571	1,33 (b)
Caspase recruitment domain family memer 6	CARD6	032587	2,08 (a)
Caspase 1 apoptosis related cysteine peptidase (interleukin 1 beta convertase)	CASP1	033292	-1,25 (b)
Caspase 1 apoptosis related cysteine peptidase	CASP5	004347	-1,36 (b)
Caspase 1 apoptosis related cysteine peptidase	CASP8	001228	-1,33 (b)
Chemokyna (c-c motive) ligand 2	CCL2	002982	-1,05(b)
Chemokyna (c-c motive) ligand 7	CCL7	006273	2,49 (a)
Cd 40 ligand	CD40LG	000074	2,72 (a)
Casp8 and fad dlike apoptosis regulator	CFLAR	003879	-1,34 (b)
Conserved helix loop helix ubiquitose kinase	CKUK	001278	-1,95 (b)
Class ii maior histocompatibility complex trans activator	CIITA	000246	-2,09 (a)
Cathepsin b	CTSB	001908	1,25 (b)
Chemokine (cxc motif) ligand 1 (melanome growth stimolating activity alpha)	CXCL1	001511	2,34 (a)
Chemokine (cxc motif) ligand 2	CXCL2	002089	1,31 (b)
Fas (tnfrsf6) asociated via death domain	FAD D	003824	1,02 (b)
Heat shock protein 90 kda alpha (citosolyc) class a member 1	HSP90AA1	001017963	-1,94 (b)
Interferon beta 1 fibrobalst	IFNB1	002176	-1,58 (b)
Interferon gamma	IFNG	000919	-1,40 (b)
Inibitor of kappa light polypeptide gene	IKBKB	001556	-1,48 (b)

Enancher in b-cell kinase beta			
Inibitor of kappa light polypeptide gene Enancher in b-cell kinase gamma	IKBKG	003639	-1,23 (b)
Inerleukine 12a (natuarl killer cell stimolatory factor 1 cytotoxic limphocyta maturation factor 1 p35)	IL12A	000882	2,52 (a)
Inerleukine 12b (natuarl killer cell stimolatory Factor 2 cytotoxic limphocyta maturation factor 2 p40)	IL 12B	002187	1,33 (b)
Interlukine 18 (interferon gamma inducuing factor)	IL18	001562	-1,08 (b)
Interleukine 1 beta	IL1B	000576	1,58 (b)
Interleukine 33	IL33	033439	1,33 (b)
Interluekine 6 (interferone beta 2)	IL 6	000600	-2,98 (a)
Interferone regulaotory factor 2	IRF2	002199	1,14 (b)
Mitogen activated protein kinase kinase kinase 7	MAP3K7	003188	1,09 (b)
Mitogen activated protein kinase 1	MAPK1	002745	-1,29 (b)
Mitogen activated protein kinase 11	MAPK11	002751	-1,11 (b)
Mitogen activated protein kinase 12	MAPK12	002969	-1,18 (b)
Mitogen activated protein kinase 13	MAPK13	002754	-1,07 (b)
Mitogen activated protein kinase 3	MAPK3	002746	-1,51 (b)
Mitogen activated protein kinase8	MAPK8	002750	1,05 (b)
Mitogen activated protein kinase 9	MAPK9	002752	1,65 (b)
Myeloid differentation primary response gene (88)	MYD88	002468	-1,02 (b)
Nuclear factor of kappa light polypeptides Gene enhancer in b-cells 1	NFKB1	003998	1,36 (b)
Nuclear factor of kappa light polypeptides Gene enhancer in b-cells inhibitor alpha	NFKBIA	020529	-1,04 (b)
Nuclear factor of kappa light polypeptides Gene enhancer in b-cells inhibitor beta	NFKBIB	002503	-1,30 (b)
Nrl family card domain	NLRC4	021209	1,61 (b)

containing 4			
Nrl family card domain containing 5	NLRC5	032206	-1,70 (b)
Nrl family pyrin domain containing 1	NLRP1	033004	-1,53 (b)
Nrl family pyrin domain containing 12	NLRP12	033297	1,53 (b)
Nrl family pyrin domain containing 3	NLRP3	183395	1,45 (b)
Nrl family pyrin domain containing 4	NLRP4	134444	1,33 (b)
Nrl family pyrin domain containing 5	NLRP5	153447	1,33 (b)
Nrl family pyrin domain containing 6	NLRP6	138329	-1,48 (b)
Nrl family x 1	NLRX1	024618	-1,75 (b)
Nucleotide binding oligomerization Domain containing 1	NOD1	006092	-1,20 (b)
Nucleotide binding oligomerization Domain containing 2	NOD2	022162	-1,36 (b)
Purinergic receptor p2x ligand gated ion channel 7	P2RX7	002562	1,65 (b)
Pannexin 1	PANX1	015368	1,09 (b)
Phosphoprotein enriched in astrocites 15	PEA15	003768	-1,16 (b)
Prolin serin threonine phosphatase Interacting protein 1	PSTPIP1	003978	1,52 (b)
Prostaglandin endoperoxidase synthase 2 (prostaglandin g/h synthase and cyclooxigenase)	PTGS2	000963	-1,08 (b)
Pyd and card domain containing	PYCARD	013258	2,32 (a)
Pyd (pyrin domain) conteing 1	PYCD1	152901	1,33 (b)
Receptor interacting serin threonine kinase 2	RIPK2	003821	1,11 (b)
Sgt1 suppressor of g2 allele of skp 1 (*s. Cerevisiae*)	SUGT1	006704	1,27 (b)
Tgf beta activated kinase 1/map 3k7 binding protein 1	TAB1	006116	-1,15 (b)
Tgf beta activated kinase 1/map 3k7 binding protein 2	TAB2	015093	1,33 (b)
Tool interleukine 1 receptor (tir) domain containing adaptor protein)	TIRAP	001039661	-1,39 (b)
Tumor necrosis factor	TNF	000594	1,83 (b)

Tumor necrosis factor (ligand) superfamily member 11	TNFSF11	003701	1,19 (b)
Tumor necrosis factor (ligand) superfamily member 14	TNFSF14	003807	1,32 (b)
Tumor necrosis factor (ligand) superfamily member 4	TNFS4	003326	-1,80 (b)
Tnf receptor associated factor 6	TRAF 6	004620	-1,03 (b)
Tioredoxine intercating protein	TXNPI	006472	-1,03 (b)

Table 3S. Fold Change between Mc Donald's meal *vs* Mc Donald's meal with not pruned vineyard red wine in Inflammasome genes.

Gene Description	Symbol	Gene Bank (NM)	Fold Change between McD and McDRW (a) *p* <0,05 (b) *p* >0,05
Absent in melanoma 2	AIM2	004833	-1,14 (b)
B-cell ccl/lynphoma 2	BCL2	000633	-1,14 (b)
Bcl2 like 1	BCL2L1	138578	-1,56 (b)
Baculoviral iap repeat containig 2	BIRC2	001166	-1,23 (b)
Baculoviral iap repeat containig 3	BIRC3	001165	-2,74 (a)
Caspase recruitment domain family memer 18	CARD18	021571	-1,02 (b)
Caspase recruitment domain family memer 6	CARD6	032587	1,80 (b)
Caspase 1 apoptosis related cysteine peptidase (interleukin 1 beta convertase)	CASP1	033292	-1,03 (b)
Caspase 1 apoptosis related cysteine peptidase	CASP5	004347	-1,77 (b)
Caspase 1 apoptosis related cysteine peptidase	CASP8	001228	1,30 (b)
Chemokyna (c-c motive) ligand 2	CCL2	002982	-2,98 (a)
Chemokyna (c-c motive) ligand 7	CCL7	006273	1,82 (b)
Cd 40 ligand	CD40LG	000074	1,64 (b)
Casp8 and fad dlike apoptosis regulator	CFLAR	003879	1,16 (b)
Conserved helix loop helix ubiquitose kinase	CKUK	001278	-1,30 (b)
Class ii maior histocompatibility complex trans activator	CIITA	000246	-1,81 (b)
Cathepsin b	CTSB	001908	1,11 (b)
Chemokine (cxc motif) ligand 1 (melanome growth stimolating activity alpha)	CXCL1	001511	2,18 (a)
Chemokine (cxc motif) ligand 2	CXCL2	002089	1,23 (b)
Fas (tnfrsf6) asociated via death domain	FAD D	003824	1,15 (b)
Heat shock protein 90 kda alpha (citosolyc) class a member 1	HSP90AA1	001017963	-1,14 (b)
Interferon beta 1 fibrobalst	IFNB1	002176	-1,46 (b)
Interferon gamma	IFNG	000919	-1,36 (b)
Inibitor of kappa light	IKBKB	001556	-1,12 (b)

polypeptide gene Enancher in b-cell kinase beta			
Inibitor of kappa light polypeptide gene Enancher in b-cell kinase gamma	IKBKG	003639	-1,13 (b)
Inerleukine 12a (natuarl killer cell stimolatory factor 1 cytotoxic limphocyta maturation factor 1 p35)	IL12A	000882	2,14 (a)
Inerleukine 12b (natuarl killer cell stimolatory Factor 2 cytotoxic limphocyta maturation factor 2 p40)	IL 12B	002187	-1,02 (b)
Interlukine 18 (interferon gamma inducuing factor)	IL18	001562	1,00 (b)
Interleukine 1 beta	IL1B	000576	1,17 (b)
Interleukine 33	IL33	033439	-1,02 (b)
Interluekine 6 (interferone beta 2)	IL 6	000600	-1,02 (b)
Interferone regulaotory factor 2	IRF2	002199	-1,11 (b)
Mitogen activated protein kinase kinase kinase 7	MAP3K7	003188	1,27 (b)
Mitogen activated protein kinase 1	MAPK1	002745	1,15 (b)
Mitogen activated protein kinase 11	MAPK11	002751	-1,02 (b)
Mitogen activated protein kinase 12	MAPK12	002969	-1,91 (b)
Mitogen activated protein kinase 13	MAPK13	002754	1,33 (b)
Mitogen activated protein kinase 3	MAPK3	002746	-1,22 (b)
Mitogen activated protein kinase8	MAPK8	002750	1,22 (b)
Mitogen activated protein kinase 9	MAPK9	002752	1,19 (b)
Myeloid differentation primary response gene (88)	MYD88	002468	1,13 (b)
Nuclear factor of kappa light polypeptides Gene enhancer in b-cells 1	NFKB1	003998	1,05 (b)
Nuclear factor of kappa light polypeptides Gene enhancer in b-cells inhibitor alpha	NFKBIA	020529	1,30 (b)
Nuclear factor of kappa light polypeptides Gene enhancer in b-cells inhibitor beta	NFKBIB	002503	1,18 (b)
Nrl family card domain	NLRC4	021209	2,12 (a)

containing 4			
Nrl family card domain containing 5	NLRC5	032206	1,04 (b)
Nrl family pyrin domain containing 1	NLRP1	033004	-1,16 (b)
Nrl family pyrin domain containing 12	NLRP12	033297	-1,37 (b)
Nrl family pyrin domain containing 3	NLRP3	183395	1,32 (b)
Nrl family pyrin domain containing 4	NLRP4	134444	-1,02 (b)
Nrl family pyrin domain containing 5	NLRP5	153447	-1,02 (b)
Nrl family pyrin domain containing 6	NLRP6	138329	1,03 (b)
Nrl family x 1	NLRX1	024618	-1,23 (b)
Nucleotide binding oligomerization Domain containing 1	NOD1	006092	1,20 (b)
Nucleotide binding oligomerization Domain containing 2	NOD2	022162	-1,43 (b)
Purinergic receptor p2x ligand gated ion channel 7	P2RX7	002562	1,75 (b)
Pannexin 1	PANX1	015368	1,80 (b)
Phosphoprotein enriched in astrocites 15	PEA15	003768	-1,25 (b)
Prolin serin threonine phosphatase Interacting protein 1	PSTPIP1	003978	1,43 (b)
Prostaglandin endoperoxidase synthase 2 (prostaglandin g/h synthase and cyclooxigenase)	PTGS2	000963	1,52 (b)
Pyd and card domain containing	PYCARD	013258	1,06 (b)
Pyd (pyrin domain) conteing 1	PYCD1	152901	-1,02 (b)
Receptor interacting serin threonine kinase 2	RIPK2	003821	1,45 (b)
Sgt1 suppressor of g2 allele of skp 1 (*s. Cerevisiae*)	SUGT1	006704	1,28 (b)
Tgf beta activated kinase 1/map 3k7 binding protein 1	TAB1	006116	1,39 (b)
Tgf beta activated kinase 1/map 3k7 binding protein 2	TAB2	015093	1,48 (b)
Tool interleukine 1 receptor (tir) domain containing adaptor protein)	TIRAP	001039661	-1,43 (b)
Tumor necrosis factor	TNF	000594	1,05 (b)
Tumor necrosis factor	TNFSF11	003701	-1,45 (b)

(ligand) superfamily member 11			
Tumor necrosis factor (ligand) superfamily member 14	TNFSF14	003807	1,12 (b)
Tumor necrosis factor (ligand) superfamily member 4	TNFS4	003326	-1,50 (b)
Tnf receptor associated factor 6	TRAF 6	004620	1,24 (b)
Tioredoxine intercating protein	TXNPI	006472	1,81 (b)

Table 1S. Fold Change between Baseline *vs* Mc Donald's meal in Oxidative Stress genes

Gene Description	Symbol	Gene Bank (NM)	Fold Change between Baseline and McD (a) *p* <0,05 (b) *p* >0,05
Albumin	ALB	000477	-2,43 (a)
Arachidonate 12 lipoxygenase	ALO12	000697	1,51 (b)
Aldheyde oxidase 1	AOX1	001159	-2,43 (a)
Apoliprotein - e	APOE	000041	-2,43 (a)
Atx1 antioxidant protein 1 omolog (yeast)	ATOX1	004045	-1,16 (b)
BCL2_ADENOVIRUS e1b19kda ITERACRTING PROTEIN 3	BNIP3	004052	1,21 (b)
Catalase	CAT	001752	1,48 (b)
Copper chaperone for superoxide dismutase	CCL5	002985	-2,60 (a)
Cytocrome b-245 beta polypeptide	CYBB	000397	1,04 (b)
Cytoglobin	CYGB	134268	-1,21 (b)
24 dehydrocholesterol redusctase	DHCR24	014762	-1,59 (b)
Dual oxidase 1	DUOX1	175940	1,88 (b)
Dual specifity phosphatase 1	DUSPO1	004417	1,42 (b)
Epoxide hydrolase 2 citoplasmic	EPHX2	001979	1,53 (b)
Eosinophyl peroxidase	EPX	000502	1,79 (b)
Forkhead box m1	FOXM1	021953	-1,22 (b)
Ferrtin heavy polypeptide 1	FTH1	002032	-1,75 (b)
Glutammate cisteine ligase, catalytic subunit	GCL2	001498	-1,30 (b)
Glutammate cisteine ligase, modifier subunit	GCLM	002061	-1,01 (b)
Glutathione peroxidase 1	GPX1	000581	1,09 (b)
Glutathione peroxidase 1 (gastrointestinal)	GPX2	002083	1,23 (b)
Glutathione peroxidase (plasma)	GPX3	002084	1,59 (b)
Glutathione peroxidase (phospholipid hydroperoxidase)	GPX4	002085	1,36 (b)
Glutathione peroxidase 5 (epididymal androgen-related protein)	GPX5	001509	-2,43 (a)
Glutathione peroxidase (olfactory)	GPX 6	182701	-2,43 (a)
Glutathione peroxidase 7	GPX7	015696	-1,64 (b)
Glutathione synthetase	GSS	000178	-1,27 (b)
Glutathione s-transpherase pi 1	GSTP1	000852	-1,35 (b)
Glutathione traspherase zeta 1	GSTZ1	001513	-1,12 (b)
General transcription factor ii i	GTF2I	001518	1,11 (b)
Eme oxigenase (decycling) 1	HMOX1	002133	-1,26 (b)
Heat shock 70 kda protein 1a	HSPA1A	005345	1,42 (b)
Keratin	KRT1	006121	1,15 (b)
Lactoperoxidase	LPO	006151	-2,43 (a)
Myoglobin	MB	005368	-2,43 (a)

Mannose-binding electin (protein c) 2 soluble	MBL2	000242	-2,19 (a)
Microsomal glutathione s-transferase 3	MGST3	004528	-1,26 (b)
Mieloperoxidase	MPO	000250	-1,09 (b)
Mpv17 mytochondrial inner membrane protein	MPV17	002437	-1,15 (b)
Methionine sulfoxide reductase a	MSRA	012331	-1,42 (b)
Metallo thionein 3	MT3	005954	-2,43 (a)
Neutrphyl cytosolic factor 1	NCF1	000265	1,31 (b)
Neutrphyl cytosolic factor 2	NCF2	000433	-1,26 (b)
Nitric oxide synthase 2 inducible	NOS2	000625	1,21 (b)
Nadph oxidase 4	NOX4	016931	-2,36 (a)
Nadph oxidase ef-hand calcium binding domani 5	NOX5	024505	-2,43 (a)
Nadph dehydrogenase quinone 1	NQO1	000903	-1,29 (b)
Nudix (nucleoside dyphosophate linked moiety x)type motif 1	NUDT1	002452	1,42 (b)
Oxidation resistance 1	OXR1	181354	1,20 (b)
Oxidative stress responsive 1	OXSR1	005109	-1,70 (b)
Pdz end lim domain 1	PDLIM1	020992	-1,35 (b)
Polynuclotide kinase 3' phosphatase	PNKB	007254	1,20 (b)
Peroxiredoxin 1	PRDX1	002574	-1,26 (b)
Peroxiredoxin 2	PRDX2	005809	-1,20 (b)
Peroxiredoxin 3	PRDX3	006793	1,09 (b)
Peroxiredoxin 4	PRDX4	006406	-1,78 (b)
Peroxiredoxin 5	PRDX5	181652	-1,63 (b)
Peroxiredoxin 6	PRDX6	004905	-1,09 (b)
Phosphatidylinositol 3,4,5 triphosphate dependent rac exchange factor 1	PREX1	020820	1,04 (b)
Prion protein	PRNP	183079	1,22 (b)
Prostaglandin-endoperoxide synthase 1 (prostaglandin g/h synthase and cyclooxigenase	PTGS1	000962	1,10 (b)
Prostaglandin-endoperoxide synthase 2 (prostaglandin g/h synthase and cyclooxigenase	PTGS2	000963	1,33 (b)
Peroxidasin omolog (drosophyla)	PXDN	012293	1,03 (b)
Ring finger protein 7	RNF7	014245	1,28 (b)
Scavenger receptro class a member 3	SCARA 3	182826	-2,43 (a)
Seleno protein s	SELS	203472	-1,09 (b)
Seleno protein p, plasma,1	SEPP1	005410	1,01 (b)
Sulfactant protein d	SFTPD	003019	-1,83 (b)
Sirtuin 2	SIRT 2	012237	-1,07 (b)
Superoxide dismustase 1 soluble	SOD 1	000454	-2,22 (a)
Superoxide dismustase 2	SOD 2	000636	-1,03 (b)

mitochondrial			
Superoxide dismustase 3 extracellular	SOD 3	003102	-2,43 (a)
Sequestosome 1	SQSTM1	003900	1,07 (b)
Sulfiredoxine 1	SRXN1	080725	1,56 (b)
Serina/treonina kinase 25	STK25	006374	1,44 (b)
Thyroid peroxidase	TPO	000547	-2,00 (a)
Titin	TTN	003319	1,05 (b)
Thioredoxine	TXN	003329	-2,43 (a)
Thioredoxine reductase 1	TXNRD1	003330	-1,02 (b)
Thioredoxine reductase 2	TXNRD2	006440	-1,01(b)
Uncoping protein 2 (mithocondrial proton carrier)	UCP2	003355	-2,22 (a)

Table 2S. Fold Change between Baseline *vs* Mc Donald's meal with not pruned vineyard Red Wine, in Oxidative Stress genes.

Gene Description	Symbol	Gene Bank (NM)	Fold Change between Baseline and McDRW (a) *p* <0,05 (b) *p* >0,05
Albumin	ALB	000477	-1,40 (b)
Arachidonate 12 lipoxygenase	ALO12	000697	1,04 (b)
Aldheyde oxidase 1	AOX1	001159	-1,40 (b)
Apoliprotein - e	APOE	000041	-1,40 (b)
Atx1 antioxidant protein 1 omolog (yeast)	ATOX1	004045	1,14 (b)
BCL2_ADENOVIRUS e1b19kda ITERACRTING PROTEIN 3	BNIP3	004052	1,07(b)
Catalase	CAT	001752	1,05(b)
Copper chaperone for superoxide dismutase	CCL5	002985	-1,25 (b)
Cytocrome b-245 beta polypeptide	CYBB	000397	-1,29 (b)
Cytoglobin	CYGB	134268	-1,08 (b)
24 dehydrocholesterol redusctase	DHCR24	014762	-1,51 (b)
Dual oxidase 1	DUOX1	175940	1,17 (b)
Dual specifity phosphatase 1	DUSPO1	004417	1,05 (b)
Epoxide hydrolase 2 citoplasmic	EPHX2	001979	1,02 (b)
Eosinophyl peroxidase	EPX	000502	1,99 (b)
Forkhead box m1	FOXM1	021953	-2,10
Ferrtin heavy polypeptide 1	FTH1	002032	1,41 (b)
Glutammate cisteine ligase, catalytic subunit	GCL2	001498	-1,41 (b)
Glutammate cisteine ligase, modifier subunit	GCLM	002061	1,25 (b)
Glutathione peroxidase 1	GPX1	000581	1,37 (b)
Glutathione peroxidase 1 (gastrointestinal)	GPX2	002083	1,32 (b)
Glutathione peroxidase (plasma)	GPX3	002084	-1,15 (b)
Glutathione peroxidase (phospholipid hydroperoxidase)	GPX4	002085	1,32 (b)
Glutathione peroxidase 5 (epididymal androgen-related protein)	GPX5	001509	-1,40 (b)
Glutathione peroxidase (olfactory)	GPX 6	182701	-1,40 (b)
Glutathione peroxidase 7	GPX7	015696	1,04 (b)
Glutathione synthetase	GSS	000178	1,46 (b)
Glutathione s-transpherase pi 1	GSTP1	000852	1,55 (b)
Glutathione traspherase zeta 1	GSTZ1	001513	1,33 (b)
GENERAL TRANSCRIPTION FACTOR II i	GTF2I	001518	1,29 (b)
Eme oxigenase (decycling) 1	HMOX1	002133	1,04 (b)
Heat shock 70 kda protein 1a	HSPA1A	005345	1,56 (b)

Keratin	KRT1	006121	-1,01 (b)
Lactoperoxidase	LPO	006151	-1,40 (b)
Myoglobin	MB	005368	-1,40 (b)
Mannose-binding electin (protein c) 2 soluble	MBL2	000242	-1,81 (b)
Microsomal glutathione s-transferase 3	MGST3	004528	1,27 (b)
Mieloperoxidase	MPO	000250	1,31 (b)
Mpv17 mytochondrial inner membrane protein	MPV17	002437	1,22 (b)
Methionine sulfoxide reductase a	MSRA	012331	1,34 (b)
Metallo thionein 3	MT3	005954	-1,40 (b)
Neutrphyl cytosolic factor 1	NCF1	000265	1,24 (b)
Neutrphyl cytosolic factor 2	NCF2	000433	1,30 (b)
Nitric oxide synthase 2 inducible	NOS2	000625	-1,25 (b)
Nadph oxidase 4	NOX4	016931	-1,40 (b)
Nadph oxidase ef-hand calcium binding domani 5	NOX5	024505	-1,40 (b)
Nadph dehydrogenase quinone 1	NQO1	000903	-1,09 (b)
Nudix (nucleoside dyphosophate linked moiety x)type motif 1	NUDT1	002452	1,24 (b)
Oxidation resistance 1	OXR1	181354	-1,55 (b)
Oxidative stress responsive 1	OXSR1	005109	1,10 (b)
Pdz end lim domain 1	PDLIM1	020992	1,42 (b)
Polynuclotide kinase 3' phosphatase	PNKB	007254	1,10 (b)
Peroxiredoxin 1	PRDX1	002574	1,02 (b)
Peroxiredoxin 2	PRDX2	005809	1,01 (b)
Peroxiredoxin 3	PRDX3	006793	-1,01 (b)
Peroxiredoxin 4	PRDX4	006406	1,00 (b)
Peroxiredoxin 5	PRDX5	181652	1,10 (b)
Peroxiredoxin 6	PRDX6	004905	-1,03 (b)
Phosphatidylinositol 3,4,5 triphosphate dependent rac exchange factor 1	PREX1	020820	-1,17 (b)
Prion protein	PRNP	183079	1,09 (b)
Prostaglandin-endoperoxide synthase 1 (prostaglandin g/h synthase and cyclooxigenase	PTGS1	000962	1,09 (b)
Prostaglandin-endoperoxide synthase 2 (prostaglandin g/h synthase and cyclooxigenase	PTGS2	000963	1,02 (b)
Peroxidasin omolog (drosophyla)	PXDN	012293	-1,40 (b)
Ring finger protein 7	RNF7	014245	1,42 (b)
Scavenger receptro class a member 3	SCARA 3	182826	-1,40 (b)
Seleno protein s	SELS	203472	1,10 (b)
Seleno protein p, plasma,1	SEPP1	005410	-1,58 (b)
Sulfactant protein d	SFTPD	003019	-1,59 (b)

Sirtuin 2	SIRT 2	012237	1,29 (b)
Superoxide dismustase 1 soluble	SOD 1	000454	-1,31 (b)
Superoxide dismustase 2 mitochondrial	SOD 2	000636	1,17 (b)
Superoxide dismustase 3 extracellular	SOD 3	003102	-1,40 (b)
Sequestosome 1	SQSTM1	003900	1,25 (b)
Sulfiredoxine 1	SRXN1	080725	-1,01 (b)
Serina/treonina kinase 25	STK25	006374	1,26 (b)
Thyroid peroxidase	TPO	000547	-1,27 (b)
Titin	TTN	003319	-1,20 (b)
Thioredoxine	TXN	003329	-2,23 (a)
Thioredoxine reductase 1	TXNRD1	003330	1,17 (b)
Thioredoxine reductase 2	TXNRD2	006440	-1,39 (b)
Uncoping protein 2 (mithocondrial proton carrier)	UCP2	003355	1,28 (b)

Table 3S. Fold Change between Mc Donald's meal *vs* Mc Donald's meal with not pruned vineyard red wine, in Oxidative Stress genes.

Gene Description	Symbol	Gene Bank (NM)	Fold Change between McD and McDRW (a) p <0,05 (b) p >0,05
Albumin	ALB	000477	1,73 (b)
Arachidonate 12 lipoxygenase	ALO12	000697	-1,45 (b)
Aldheyde oxidase 1	AOX1	001159	1,73 (b)
Apoliprotein - e	APOE	000041	1,73 (b)
Atx1 antioxidant protein 1 omolog (yeast)	ATOX1	004045	1,32 (b)
BCL2_ADENOVIRUS e1b19kda ITERACRTING PROTEIN 3	BNIP3	004052	-1,13 (b)
Catalase	CAT	001752	-1,40 (b)
Copper chaperone for superoxide dismutase	CCL5	002985	2,08 (a)
Cytocrome b-245 beta polypeptide	CYBB	000397	-1,34 (b)
Cytoglobin	CYGB	134268	1,13 (b)
24 dehydrocholesterol redusctase	DHCR24	014762	1,05 (b)
Dual oxidase 1	DUOX1	175940	-1,60 (b)
Dual specifity phosphatase 1	DUSPO1	004417	-1,35 (b)
Epoxide hydrolase 2 citoplasmic	EPHX2	001979	-1,49 (b)
Eosinophyl peroxidase	EPX	000502	1,11 (b)
Forkhead box m1	FOXM1	021953	-1,72 (b)
Ferrtin heavy polypeptide 1	FTH1	002032	2,46 (a)
Glutammate cisteine ligase, catalytic subunit	GCL2	001498	-1,09 (b)
Glutammate cisteine ligase, modifier subunit	GCLM	002061	1,26 (b)
Glutathione peroxidase 1	GPX1	000581	1,26 (b)
Glutathione peroxidase 1 (gastrointestinal)	GPX2	002083	1,07 (b)
Glutathione peroxidase (plasma)	GPX3	002084	-1,83 (b)
Glutathione peroxidase (phospholipid hydroperoxidase)	GPX4	002085	-1,03 (b)
Glutathione peroxidase 5 (epididymal androgen-related protein)	GPX5	001509	1,73 (b)
Glutathione peroxidase (olfactory)	GPX 6	182701	1,73 (b)
Glutathione peroxidase 7	GPX7	015696	1,72 (b)
Glutathione synthetase	GSS	000178	1,85 (b)
Glutathione s-transpherase pi 1	GSTP1	000852	2,08 (a)
Glutathione traspherase zeta 1	GSTZ1	001513	1,48 (b)
GENERAL TRANSCRIPTION FACTOR II i	GTF2I	001518	1,16 (b)
Eme oxigenase (decycling) 1	HMOX1	002133	1,30 (b)
Heat shock 70 kda protein 1a	HSPA1A	005345	1,10 (b)
Keratin	KRT1	006121	-1,16 (b)

Lactoperoxidase	LPO	006151	1,73 (b)
Myoglobin	MB	005368	1,73 (a)
Mannose-binding electin (protein c) 2 soluble	MBL2	000242	1,21 (b)
Microsomal glutathione s-transferase 3	MGST3	004528	1,59 (b)
Mieloperoxidase	MPO	000250	1,43 (b)
Mpv17 mytochondrial inner membrane protein	MPV17	002437	1,40 (b)
Methionine sulfoxide reductase a	MSRA	012331	1,91 (b)
Metallo thionein 3	MT3	005954	1,73 (b)
Neutrphyl cytosolic factor 1	NCF1	000265	-1,06 (b)
Neutrphyl cytosolic factor 2	NCF2	000433	1,64 (b)
Nitric oxide synthase 2 inducible	NOS2	000625	-1,51 (b)
Nadph oxidase 4	NOX4	016931	1,68 (b)
Nadph oxidase ef-hand calcium binding domani 5	NOX5	024505	1,73 (b)
Nadph dehydrogenase quinone 1	NQO1	000903	1,19 (b)
Nudix (nucleoside dyphosophate linked moiety x)type motif 1	NUDT1	002452	-1,14 (b)
Oxidation resistance 1	OXR1	181354	-1,85 (b)
Oxidative stress responsive 1	OXSR1	005109	1,88 (b)
Pdz end lim domain 1	PDLIM1	020992	1,91 (b)
Polynuclotide kinase 3' phosphatase	PNKB	007254	-1,09 (b)
Peroxiredoxin 1	PRDX1	002574	1,28 (b)
Peroxiredoxin 2	PRDX2	005809	1,21 (b)
Peroxiredoxin 3	PRDX3	006793	-1,10 (b)
Peroxiredoxin 4	PRDX4	006406	1,78 (b)
Peroxiredoxin 5	PRDX5	181652	1,80 (b)
Peroxiredoxin 6	PRDX6	004905	1,05 (b)
Phosphatidylinositol 3,4,5 triphosphate dependent rac exchange factor 1	PREX1	020820	-1,22 (b)
Prion protein	PRNP	183079	-1,12 (b)
Prostaglandin-endoperoxide synthase 1 (prostaglandin g/h synthase and cyclooxigenase	PTGS1	000962	-1,01 (b)
Prostaglandin-endoperoxide synthase 2 (prostaglandin g/h synthase and cyclooxigenase	PTGS2	000963	-1,30 (b)
Peroxidasin omolog (drosophyla)	PXDN	012293	-1,44 (b)
Ring finger protein 7	RNF7	014245	1,11 (b)
Scavenger receptro class a member 3	SCARA 3	182826	1,73 (b)
Seleno protein s	SELS	203472	1,20 (b)
Seleno protein p, plasma,1	SEPP1	005410	-1,59 (b)
Sulfactant protein d	SFTPD	003019	1,15 (b)
Sirtuin 2	SIRT 2	012237	1,39 (b)

Superoxide dismustase 1 soluble	SOD 1	000454	1,69 (b)
Superoxide dismustase 2 mitochondrial	SOD 2	000636	1,20 (b)
Superoxide dismustase 3 extracellular	SOD 3	003102	1,73 (b)
Sequestosome 1	SQSTM1	003900	1,17 (b)
Sulfiredoxine 1	SRXN1	080725	-1,57 (b)
Serina/treonina kinase 25	STK25	006374	-1,14 (b)
Thyroid peroxidase	TPO	000547	1,57 (b)
Titin	TTN	003319	-1,26 (b)
Thioredoxine	TXN	003329	1,09 (b)
Thioredoxine reductase 1	TXNRD1	003330	1,19 (b)
Thioredoxine reductase 2	TXNRD2	006440	-1,38 (b)
Uncoping protein 2 (mithocondrial proton carrier)	UCP2	003355	2,83 (a)

Table 1S. Fold Change between Baseline and Mc Donald's meal, in Human Drug Metabolism

Gene Description	Symbol	Gene Bank (NM)	Fold Change between Baseline and McD (a) *p* <0,05 (b) *p* >0,05
Atp binding cassette sub family c (cftr/mrp) member 1	ABCC1	004996	1,54 (b)
Amiloride binding protein 1 (amine oxidase (copper containing))	ABP1	001091	-1,30 (b)
Alchol dehydrogenase 1 c (classe 1) gamma polypeptide	ADH1C	000669	-1,61 (b)
Alchol dehydrogenase 4 classe 2 pi polypeptide	ADH4	000670	2,15 (a)
Alchol dehydrogenase 5 classe 3 ch i polypeptide	ADH5	000671	-1,44 (b)
Alchol dehydrogenase 6 (classe 5)	ADH6	000672	1,12 (b)
Aryl hydrocarbon receptro	AHR	001621	-1,33 (b)
Aminolevulinate dehydratase	ALA D	000031	-1,60 (b)
Aldhyde dehydrogenase 1 family member a1	ALDH1A1	000689	-2,28 (a)
Arachidonate 15-lipoxygenase	ALOX15	001140	2,02 (a)
Arachidonate 5-lipoxygenase	ALOX5	000698	-2,02 (a)
Apoliprotein - e	APOE	000041	2,23 (a)
Aril hydrocarbon receptor nuclear translocator	ARNT	001668	-1,00 (b)
Arsa arsenite transporter atp binding omolog 1 (bacterial)	ANSA1	004317	1,25 (b)
Biliverdin reductase a	DLDRA	000712	1,10 (b)
Biliverdin reductase b (flavinreductase nadph)	DLDRB	000713	-1,32 (b)
Carboxylesterase 2	CES 2	198061	-1,70 (b)
Carboxilesterase 1 pseudogene 1	CES1P1	003276	1,73 (b)
Carbohydrate (keratan sulphate gal-6) sulphotransferase 1	CHST1	003654	-1,70 (b)
Cytocrome p450 family 11 subfamily b polypeptide 2	CYP11B2	000498	-1,70 (b)
Cytocrome p450 family 17 subfamily a polypeptide1	CYP17A1	000102	-1,70 (b)
Cytocrome p450 family 19 subfamily a polypeptide 1	CYP19A1	000103	-1,70 (b)
Cytocrome p450 family 1 subfamily a polypeptide 1	CYP1A1	000499	-1,70 (b)
Cytocrome p450 family 2 subfamily b polypeptide 6	CYP2B6	000767	-1,70 (b)
Cytocrome p450 family 2 subfamily c polypeptide 8	CYP2C8	000770	-1,70 (b)
Cytocrome p450 family 2 subfamily c polypeptide 9	CYP2C9	000771	-1,70 (b)
Cytocrome p450 family 2 subfamily d polypeptide 6	CYP2D6	000106	1,30 (b)
Cytocrome p450 family 2	CYP2E1	000773	1,34 (b)

subfamily e polypeptide 1			
Cytocrome p450 family 2 subfamily f polypeptide 1	CYP2F1	000774	-1,70 (b)
Cytocrome p450 family 2 subfamily j polypeptide 2	CY2J2	000775	1,11 (b)
Cytocrome b5 reductase 3	CYB5R3	007326	-1,65 (b)
Epoxide hydorlase 1 microsomal (xenobiotic)	EPHX1	000120	-1,37 (b)
Fat acide hydrolase	FAAH	001441	1,09 (b)
Fructose 1,6 bisphosphatase 1	FBP1	000507	-1,62 (b)
Glutamate decarboxylase 1 (brain 67 kda)	GAD1	000817	-1,70 (b)
Glucokinase (exokinase) regulator	GCKR	001486	1,36 (b)
Gamma glutamil tranferase 1	GGT1	005265	1,14 (b)
Glucose 6 phosphate isomerase	GPI	000175	-1,56 (b)
Glutathione peroxidase 1 (gastrointestinal)	GPX2	000581	1,42 (b)
Glutathione peroxidase (plasma)	GPX3	002083	-1,48 (b)
Glutathione peroxidase 5 (epididymal androgen-related protein)	GPX5	002085	-1,70 (b)
Glutathione s-transferase alpha 4	GSTA4	001512	1,51 (b)
Glutathione s-transferase mu 3 (brain)	GSTM3	000849	-1,64 (b)
Glutathione s-transferase mu 5	GSTM5	000851	-1,03 (b)
Glutathione s-transferase p 1	GSTP1	000852	-1,20 (b)
Glutathione s-transferase t 1	GSTT1	000853	1,48 (b)
Glutathione s-transferase z 1	GSTZ1	001513	1,19 (b)
Hydroxisteroid (17 beta) dehydorgenase 2	HSD17 B2	002153	-2,26 (a)
Hydroxisteroid (17 beta) dehydorgenase 3	HSD17 B3	000197	1,58 (b)
Lactoperoxidase	LPO	006151	1,73 (b)
Myristoidated alanine rich protein kinase c substrate	MARCKS	002356	1,49 (b)
Microsomal glutathione s-tranferase 1	MGST1	0203000	-1,03 (b)
Microsomal glutathione s-tranferase 2	MGST2	002413	-1,39 (b)
Microsomal glutathione s-tranferase 3	MGST3	004528	-1,08 (b)
Myeloperoxidase	MPO	000250	-1,86 (b)
Metallotioneine 2a	MT2A	005953	-2,28 (a)
Metallotioneine 3	MT3	005954	1,53 (b)
Methileneteraydropholate reductase (nadph)	MTHFR	005957	1,23 (b)
N-acetiltransferase 1 (arilamina n-acetiltrasnferase)	NAT1	000662	-1,61 (b)
N-acetiltransferase 2 (arilamina n-acetiltrasnferase)	NAT2	000015	-1,62 (b)
Nitric oxide synthase 3 (endotelial cell)	NOS3	000603	-1,93 (b)
Nadph dehydrogenase quinone 1	NQO1	000903	-1,07 (b)

Piruvate kinase liver and rbc	PKLR	000298	-1,35 (b)
Piruvate kinase (muscle)	PKM2	002654	1,04 (b)
Paraoxonase 1	PON1	000446	-1,70 (b)
Paraoxonase 2	PON2	000305	1,27 (b)
Paraoxonase 3	PON3	000940	1,65 (b)
Swi-snf related matrix associated actin dependent regulator of cromatin subfamily a-like 1	SMARCAL 1	014140	1,13 (b)
Stannin	SNN	003498	1,29 (b)
Steroid 5 alpha reductase alpha polipeptide 1 (3-oxo-5-alpha-steroid delta 4-dehydrogenase alpha1)	SRD5A1	001047	1,02 (b)
Steroid 5 alpha reductase alpha polipeptide 2 (3-oxo-5-alpha-steroid delta 4-dehydrogenase alpha2)	SRD5A2	000348	-1,70 (b)

Table 2S. Fold Change between Baseline *vs* Mc Donald's meal with Not pruned vineyard red wine, in Oxidative Stress genes.

Gene Description	Symbol	Gene Bank (NM)	Fold Change between Baseline and McDRW (a) *p* <0,05 (b) *p* >0,05
Atp binding cassette sub family c (cftr/mrp) member 1	ABCC1	004996	1,43 (b)
Amiloride binding protein 1 (amine oxidase (copper containing))	ABP1	001091	-1,97 (b)
Alchol dehydrogenase 1 c (classe 1) gamma polypeptide	ADH1C	000669	-1,26 (b)
Alchol dehydrogenase 4 classe 2 pi polypeptide	ADH4	000670	-1,16 (b)
Alchol dehydrogenase 5 classe 3 ch i polypeptide	ADH5	000671	-1,50 (b)
Alchol dehydrogenase 6 (classe 5)	ADH6	000672	-1,41 (b)
Aryl hydrocarbon receptro	AHR	001621	2,03 (a)
Aminolevulinate dehydratase	ALA D	000031	1,08 (b)
Aldhyde dehydrogenase 1 family member a1	ALDH1A1	000689	1,00 (b)
Arachidonate 15-lipoxygenase	ALOX15	001140	1,18 (b)
Arachidonate 5-lipoxygenase	ALOX5	000698	1,31 (b)
Apoliprotein - e	APOE	000041	-1,26 (b)
Aril hydrocarbon receptor nuclear translocator	ARNT	001668	-1,42 (b)
Arsa arsenite transporter atp binding omolog 1 (bacterial)	ANSA1	004317	1,41 (b)
Biliverdin reductase a	DLDRA	000712	1,49 (b)
Biliverdin reductase b (flavinreductase nadph)	DLDRB	000713	-1,03 (b)
Carboxylesterase 2	CES 2	198061	-1,26 (b)
Carboxilesterase 1 pseudogene 1	CES1P1	003276	-1,08 (b)
Carbohydrate (keratan sulphate gal-6) sulphotransferase 1	CHST1	003654	-1,26 (b)
Cytocrome p450 family 11 subfamily b polypeptide 2	CYP11B2	000498	-1,26 (b)
Cytocrome p450 family 17 subfamily a polypeptide1	CYP17A1	000102	-1,26 (b)
Cytocrome p450 family 19 subfamily a polypeptide 1	CYP19A1	000103	-1,26 (b)
Cytocrome p450 family 1 subfamily a polypeptide 1	CYP1A1	000499	-1,26 (b)
Cytocrome p450 family 2 subfamily b polypeptide 6	CYP2B6	000767	-1,26 (b)
Cytocrome p450 family 2 subfamily c polypeptide 8	CYP2C8	000770	-1,26 (b)
Cytocrome p450 family 2 subfamily c polypeptide 9	CYP2C9	000771	-1,26 (b)

Cytocrome p450 family 2 subfamily d polypeptide 6	CYP2D6	000106	1,07 (b)
Cytocrome p450 family 2 subfamily e polypeptide 1	CYP2E1	000773	1,12 (b)
Cytocrome p450 family 2 subfamily f polypeptide 1	CYP2F1	000774	-1,26 (b)
Cytocrome p450 family 2 subfamily j polypeptide 2	CY2J2	000775	1,95 (b)
Cytocrome b5 reductase 3	CYB5R3	007326	1,13 (b)
Epoxide hydorlase 1 microsomal (xenobiotic)	EPHX1	000120	-1,25 (b)
Fat acide hydrolase	FAAH	001441	-1,18 (b)
Fructose 1,6 bisphosphatase 1	FBP1	000507	1,20 (b)
Glutamate decarboxylase 1 (brain 67 kda)	GAD1	000817	-1,26 (b)
Glucokinase (exokinase) regulator	GCKR	001486	-1,26 (b)
Gamma glutamil tranferase 1	GGT1	005265	1,04 (b)
Glucose 6 phosphate isomerase	GPI	000175	-1,07 (b)
Glutathione peroxidase 1 (gastrointestinal)	GPX2	000581	1,12 (b)
Glutathione peroxidase (plasma)	GPX3	002083	1,59 (b)
Glutathione peroxidase 5 (epididymal androgen-related protein)	GPX5	002085	-1,26 (b)
Glutathione s-transferase alpha 4	GSTA4	001512	-1,15 (b)
Glutathione s-transferase mu 3 (brain)	GSTM3	000849	1,40 (b)
Glutathione s-transferase mu 5	GSTM5	000851	-1,26 (b)
Glutathione s-transferase p 1	GSTP1	000852	1,03 (b)
Glutathione s-transferase t 1	GSTT1	000853	1,77 (b)
Glutathione s-transferase z 1	GSTZ1	001513	1,25 (b)
Hydroxisteroid (17 beta) dehydorgenase 2	HSD17 B2	002153	-1,83 (b)
Hydroxisteroid (17 beta) dehydorgenase 3	HSD17 B3	000197	-1,49 (b)
Lactoperoxidase	LPO	006151	-1,49 (b)
Myristoidated alanine rich protein kinase c substrate	MARCKS	002356	1,66 (b)
Microsomal glutathione s-tranferase 1	MGST1	0203000	1,42 (b)
Microsomal glutathione s-tranferase 2	MGST2	002413	1,11 (b)
Microsomal glutathione s-tranferase 3	MGST3	004528	1,02 (b)
Myeloperoxidase	MPO	000250	-1,26 (b)
Metallotioneine 2a	MT2A	005953	1,18 (b)
Metallotioneine 3	MT3	005954	2,06 (a)
Methileneteraydropholate reductase (nadph)	MTHFR	005957	1,65 (b)
N-acetiltransferase 1 (arilamina n-acetiltrasnferase)	NAT1	000662	1,76 (b)
N-acetiltransferase 2 (arilamina n-acetiltrasnferase)	NAT2	000015	-1,26 (b)

Nitric oxide synthase 3 (endotelial cell)	NOS3	000603	1,30 (b)
Nadph dehydrogenase quinone 1	NQO1	000903	1,14 (b)
Piruvate kinase liver and rbc	PKLR	000298	-1,26 (b)
Piruvate kinase (muscle)	PKM2	002654	-1,61 (b)
Paraoxonase 1	PON1	000446	-1,26 (b)
Paraoxonase 2	PON2	000305	-1,45 (b)
Paraoxonase 3	PON3	000940	-1,26 (b)
Swi-snf related matrix associated actin dependent regulator of cromatin subfamily a-like 1	SMARCAL 1	014140	1,08 (b)
Stannin	SNN	003498	1,09 (b)
Steroid 5 alpha reductase alpha polipeptide 1 (3-oxo-5-alpha-steroid delta 4-dehydrogenase alpha1)	SRD5A1	001047	1,22 (b)
Steroid 5 alpha reductase alpha polipeptide 2 (3-oxo-5-alpha-steroid delta 4-dehydrogenase alpha2)	SRD5A2	000348	-1,26 (b)

Table 3S. Fold Change between Mc Donald's meal *vs* Mc Donald's meal with not pruned vineyard red wine, in Human Drug Metabolism genes.

**Gene Description**	**Symbol**	**Gene Bank (NM)**	**Fold Change between McD and McDRW (a) *p* <0,05 (b) *p* >0,05**
Atp binding cassette sub family c (cftr/mrp) member 1	ABCC1	004996	-1,08 (b)
Amiloride binding protein 1 (amine oxidase (copper containing))	ABP1	001091	-1,52 (b)
Alchol dehydrogenase 1 c (classe 1) gamma polypeptide	ADH1C	000669	1,28 (b)
Alchol dehydrogenase 4 classe 2 pi polypeptide	ADH4	000670	-2,48 (a)
Alchol dehydrogenase 5 classe 3 ch i polypeptide	ADH5	000671	-1,04 (b)
Alchol dehydrogenase 6 (classe 5)	ADH6	000672	-1,58 (b)
Aryl hydrocarbon receptro	AHR	001621	2,71 (a)
Aminolevulinate dehydratase	ALA D	000031	1,73 (b)
Aldhyde dehydrogenase 1 family member a1	ALDH1A1	000689	2,28 (a)
Arachidonate 15-lipoxygenase	ALOX15	001140	-1,71 (b)
Arachidonate 5-lipoxygenase	ALOX5	000698	2,65
Apoliprotein - e	APOE	000041	-2,79
Aril hydrocarbon receptor nuclear translocator	ARNT	001668	-1,42 (b)
Arsa arsenite transporter atp binding omolog 1 (bacterial)	ANSA1	004317	1,12 (b)
Biliverdin reductase a	DLDRA	000712	1,35 (b)
Biliverdin reductase b (flavinreductase nadph)	DLDRB	000713	1,27 (b)
Carboxylesterase 2	CES 2	198061	1,35 (b)
Carboxilesterase 1 pseudogene 1	CES1P1	003276	-1,87 (b)
Carbohydrate (keratan sulphate gal-6) sulphotransferase 1	CHST1	003654	1,35 (b)
Cytocrome p450 family 11 subfamily b polypeptide 2	CYP11B2	000498	1,35 (b)
Cytocrome p450 family 17 subfamily a polypeptide1	CYP17A1	000102	1,35 (b)
Cytocrome p450 family 19 subfamily a polypeptide 1	CYP19A1	000103	1,35 (b)
Cytocrome p450 family 1 subfamily a polypeptide 1	CYP1A1	000499	1,35 (b)
Cytocrome p450 family 2 subfamily b polypeptide 6	CYP2B6	000767	1,35 (b)
Cytocrome p450 family 2 subfamily c polypeptide 8	CYP2C8	000770	1,35 (b)
Cytocrome p450 family 2 subfamily c polypeptide 9	CYP2C9	000771	1,35 (b)
Cytocrome p450 family 2 subfamily d polypeptide 6	CYP2D6	000106	-1,22 (b)

Cytocrome p450 family 2 subfamily e polypeptide 1	CYP2E1	000773	-1,20 (b)
Cytocrome p450 family 2 subfamily f polypeptide 1	CYP2F1	000774	1,35 (b)
Cytocrome p450 family 2 subfamily j polypeptide 2	CY2J2	000775	1,75 (b)
Cytocrome b5 reductase 3	CYB5R3	007326	1,88 (b)
Epoxide hydorlase 1 microsomal (xenobiotic)	EPHX1	000120	1,10 (b)
Fat acide hydrolase	FAAH	001441	-1,29 (b)
Fructose 1,6 bisphosphatase 1	FBP1	000507	1,94 (b)
Glutamate decarboxylase 1 (brain 67 kda)	GAD1	000817	1,35 (b)
Glucokinase (exokinase) regulator	GCKR	001486	-1,71 (b)
Gamma glutamil tranferase 1	GGT1	005265	-1,10 (b)
Glucose 6 phosphate isomerase	GPI	000175	1,46 (b)
Glutathione peroxidase 1 (gastrointestinal)	GPX2	000581	-1,27 (b)
Glutathione peroxidase (plasma)	GPX3	002083	2,36 (a)
Glutathione peroxidase 5 (epididymal androgen-related protein)	GPX5	002085	1,35 (b)
Glutathione s-transferase alpha 4	GSTA4	001512	-1,73 (b)
Glutathione s-transferase mu 3 (brain)	GSTM3	000849	2,29 (a)
Glutathione s-transferase mu 5	GSTM5	000851	-1,22 (b)
Glutathione s-transferase p 1	GSTP1	000852	1,24 (b)
Glutathione s-transferase t 1	GSTT1	000853	1,20 (b)
Glutathione s-transferase z 1	GSTZ1	001513	1,05 (b)
Hydroxisteroid (17 beta) dehydorgenase 2	HSD17 B2	002153	1,24 (b)
Hydroxisteroid (17 beta) dehydorgenase 3	HSD17 B3	000197	-2,37 (a)
Lactoperoxidase	LPO	006151	-2,59 (a)
Myristoidated alanine rich protein kinase c substrate	MARCKS	002356	1,12 (b)
Microsomal glutathione s-tranferase 1	MGST1	0203000	1,45 (b)
Microsomal glutathione s-tranferase 2	MGST2	002413	1,55 (b)
Microsomal glutathione s-tranferase 3	MGST3	004528	1,11 (b)
Myeloperoxidase	MPO	000250	1,48 (b)
Metallotioneine 2a	MT2A	005953	2,69 (a)
Metallotioneine 3	MT3	005954	1,35 (b)
Methileneteraydropholate reductase (nadph)	MTHFR	005957	1,35 (b)
N-acetiltransferase 1 (arilamina n-acetiltrasnferase)	NAT1	000662	2,82 (a)
N-acetiltransferase 2 (arilamina n-acetiltrasnferase)	NAT2	000015	1,29 (b)
Nitric oxide synthase 3 (endotelial cell)	NOS3	000603	2,51 (a)

Nadph dehydrogenase quinone 1	NQO1	000903	1,22 (b)
Piruvate kinase liver and rbc	PKLR	000298	1,08 (b)
Piruvate kinase (muscle)	PKM2	002654	-1,67 (b)
Paraoxonase 1	PON1	000446	1,35 (b)
Paraoxonase 2	PON2	000305	-1,84 (b)
Paraoxonase 3	PON3	000940	-2,07 (a)
Swi-snf related matrix associated actin dependent regulator of cromatin subfamily a-like 1	SMARCAL 1	014140	-1,04 (b)
Stannin	SNN	003498	-1,18 (b)
Steroid 5 alpha reductase alpha polipeptide 1 (3-oxo-5-alpha-steroid delta 4-dehydrogenase alpha1)	SRD5A1	001047	1,20 (b)
Steroid 5 alpha reductase alpha polipeptide 2 (3-oxo-5-alpha-steroid delta 4-dehydrogenase alpha2)	SRD5A2	000348	1,35 (b)
